# Hepatocyte differentiation requires anisotropic expansion of bile canaliculi

**DOI:** 10.1242/dev.202777

**Published:** 2024-11-21

**Authors:** Maarten P. Bebelman, Lenka Belicova, Elzbieta Gralinska, Tobias Jumel, Aparajita Lahree, Sarah Sommer, Andrej Shevchenko, Timofei Zatsepin, Yannis Kalaidzidis, Martin Vingron, Marino Zerial

**Affiliations:** ^1^ Max Planck Institute of Molecular Cell Biology and Genetics, 01307 Dresden, Germany; ^2^ Department of Computational Molecular Biology, Max Planck Institute for Molecular Genetics, 14195 Berlin, Germany; ^3^ Department of Chemistry, Lomonosov Moscow State University, Moscow 119991, Russia

**Keywords:** Bile canaliculi, Cell differentiation, Cell fate, Cell polarity, Liver development, Rab35, Mouse

## Abstract

During liver development, bipotential progenitor cells called hepatoblasts differentiate into hepatocytes or cholangiocytes. Hepatocyte differentiation is uniquely associated with multi-axial polarity, enabling the anisotropic expansion of apical lumina between adjacent cells and formation of a three-dimensional network of bile canaliculi. Cholangiocytes, the cells forming the bile ducts, exhibit the vectorial polarity characteristic of epithelial cells. Whether cell polarization feeds back on the gene regulatory pathways governing hepatoblast differentiation is unknown. Here, we used primary mouse hepatoblasts to investigate the contribution of anisotropic apical expansion to hepatocyte differentiation. Silencing of the small GTPase Rab35 caused isotropic lumen expansion and formation of multicellular cysts with the vectorial polarity of cholangiocytes. Gene expression profiling revealed that these cells express reduced levels of hepatocyte markers and upregulate genes associated with cholangiocyte identity. Timecourse RNA sequencing demonstrated that loss of lumen anisotropy precedes these transcriptional changes. Independent alterations in apical lumen morphology induced either by modulation of the subapical actomyosin cortex or by increased intraluminal pressure caused similar transcriptional changes. These findings suggest that cell polarity and lumen morphogenesis feed back to hepatoblast-to-hepatocyte differentiation.

## Teaser

**Highlighted Article:** Differentiation of mouse liver progenitors to functional hepatocytes requires anisotropic elongation of their nascent apical surfaces into tubular bile canaliculi.

## INTRODUCTION

Cellular differentiation and morphogenesis occur in concert to build functional tissues during development. Whereas differential gene expression determines the building blocks that shape cells and tissues, tissue morphogenesis conversely feeds back on gene regulatory pathways and impacts cell fate decisions via external chemical and mechanical cues (Tatapudy et al., 2017; Motegi et al., 2020; Chan et al., 2017). The developing liver provides an excellent example to explore the intertwined mechanisms regulating cell fate. Hepatoblasts are bipotential liver progenitors that differentiate into two functionally and morphologically distinct epithelial cell types: hepatocytes and cholangiocytes (Musch, 2018; Tanimizu and Mitaka, 2017). Hepatocytes are liver parenchymal cells with an unusual cell polarity that is unique among epithelia (Morales-Navarrete et al., 2019). In contrast to simple epithelia, which have a single apico-basal axis, hepatocytes have multiple apical (and basal) surfaces. Their apical surfaces are shared between juxtaposed hepatocytes and grow anisotropically, i.e. forming elongated tubules instead of spheres. These fine tubules, known as bile canaliculi (BC), form a network in which bile is secreted and transported to bile ducts, larger epithelial tubes formed by cholangiocytes that transfer bile to the intestine. In contrast to hepatocytes, cholangiocytes display the common vectorial polarity, whereby their apical surfaces face a common lumen, similar to epithelial tubes in other organs.

We recently described that to form BC, hepatocytes generate transversal apical connections, termed apical bulkheads, that mechanically connect the apical membranes of juxtaposed hepatocytes and enforce the anisotropic expansion of nascent apical lumina into tubular BC (Bebelman et al., 2023; Belicova et al., 2021). Loss of apical bulkheads, through genetic manipulation (see below), results in the isotropic expansion of newly formed apical lumina leading to the formation of multicellular cysts in which the cells adopt a vectorial polarization, similar to cholangiocytes.

During development, the vast majority of hepatoblasts differentiate into hepatocytes through a default differentiation programme, whereas hepatoblasts near the portal veins branch away from the default programme to differentiate into cholangiocytes by suppressing the hepatocyte-specific and activating a cholangiocyte-specific transcriptional programme (Yang et al., 2017; Prior et al., 2019; Yang et al., 2023). In mouse, the timing of hepatocyte differentiation [embryonic day (E) 14.5-E15.5] coincides with hepatoblast polarization and BC formation (Delpierre et al., 2024 preprint). How hepatoblast polarization and differentiation are linked and contribute to hepatocyte cell fate determination is an unexplored problem. Recent studies in a variety of cellular and animal model systems have revealed a functional link between the molecular mechanisms underlying cell polarization, mechanical forces at the cell and tissue level, and the genetic programme of cell lineage in epithelia (Rose and Gönczy, 2014; Lamba and Zernicka-Goetz, 2023; Ahuja and Cleaver, 2022; Motegi et al., 2020; Hashimoto and Munro, 2018; Hirate et al., 2013).

Whether the cellular polarization phenotype feeds back on the gene regulatory pathways that govern hepatoblast differentiation is currently unclear. We have recently modelled hepatocyte differentiation in the embryonic liver by *in vitro* culture of primary mouse hepatoblasts. Using this system, we identified a key regulator, the small GTPase Rab35, that plays a role in actin organization and endocytic recycling (Klinkert and Echard, 2016) and expression of which can be manipulated to switch the polarity phenotype of the differentiating cells (Belicova et al., 2021). Instead of the typical multi-axial polarization of hepatocytes, upon silencing of Rab35, hepatoblasts adopt a vectorial polarization with their apical membranes facing the central lumen, similar to cholangiocytes in bile ducts. We took advantage of this system to examine the consequence of a switch from multi-axial to vectorial polarity for the cell fate determination of hepatoblasts.

## RESULTS AND DISCUSSION

### Rab35 silencing impairs hepatocyte polarization and differentiation

**Fig. 1.**
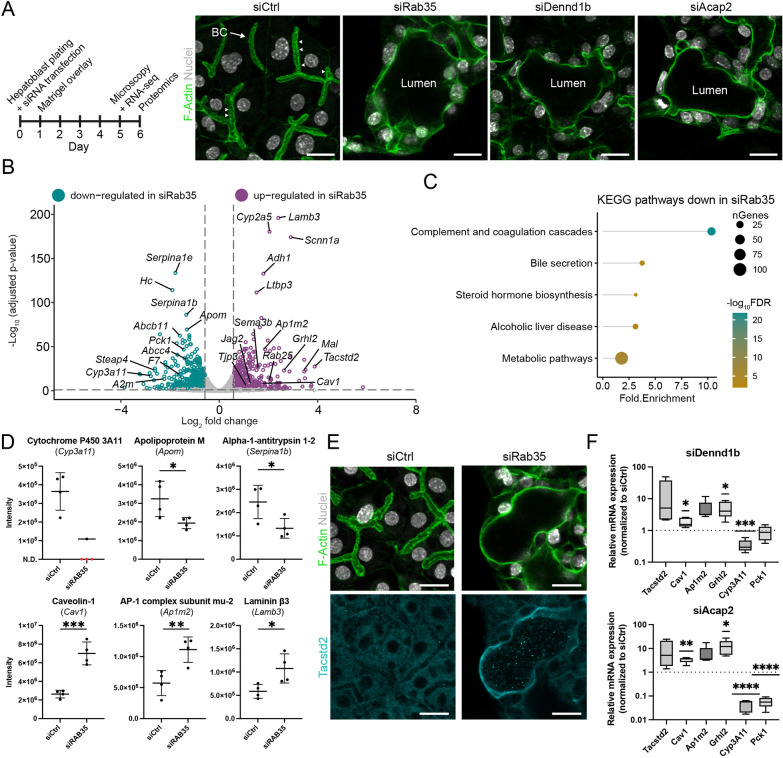
**Rab35 silencing impairs hepatocyte polarization and differentiation.** (A) Left: Experiment design. Right: *In vitro*-differentiated hepatocytes transfected with control siRNA (siCtrl) or Rab35-, Dennd1b-, Acap2-targeting siRNAs. Arrowheads highlight apical bulkheads. (B) Differentially regulated genes upon siRab35-transfection, compared to siCtrl (*n*=3). (C) Selected pathways from KEGG pathway enrichment analysis based on genes downregulated in siRab35-transfected cells. (D) Mass spectrometry-based intensities of hepatocyte- (upper) or cholangiocyte-associated (lower) proteins (*n*=4). Mean±s.d. N.D., not detected. (E) SiCtrl- or siRab35-transfected differentiating hepatoblasts. (F) qRT-PCR for genes associated with vectorial polarity and cholangiocyte identity (*Tacstd2*, *Cav1*, *Ap1m2*, *Grhl2*) and hepatocyte identity (*Cyp3a11*, *Pck1*) following siDennd1b and siAcap2 transfection (*n*=5). Whiskers represent the minimal and maximal values, the box extends from the 25th to 75th percentiles. The horizontal line in the boxes is the median. *P<0.05; **P<0.01; ***P<0.001; ****P<0.0001. See Materials and Methods for statistical analyses. Scale bars: 20 µm.

During the differentiation of primary hepatoblasts towards hepatocytes *in vitro*, nascent apical lumina between adjacent cells expand anisotropically to form elongated tubes (Fig. 1A, Fig. S1A), recapitulating BC formation *in vivo* (Belicova et al., 2021). Silencing of Rab35 results in a loss of apical bulkheads, isotropic apical lumen expansion, and, ultimately, the formation of multicellular cysts (Fig. 1A, Fig. S1A) (Belicova et al., 2021). We first verified that this phenotype is not an epiphenomenon but reflects the requirement of a molecular pathway required for apical bulkhead formation. We therefore tested whether silencing of the Rab35 guanine-nucleotide exchange factor Dennd1b, or the Rab35 effector Acap2, phenocopies the loss of Rab35. Indeed, their silencing also resulted in isotropic lumen expansion and cyst formation (Fig. 1A, Fig. S1A,B), indicating the requirement of a Rab35 pathway for hepatocyte polarity.

Given the emerging relationship between cell polarization, mechanical forces and the genetic programme of cell lineage in epithelia, the change of cell polarity caused by Rab35 knockdown raises the question of the extent to which hepatoblast-to-hepatocyte differentiation is also affected. Previously, we showed that cells in the cysts still expressed the hepatocyte marker HNF4α but were negative for the cholangiocyte marker Sox9 (Belicova et al., 2021), suggesting that they may retain their hepatocyte identity. However, to explore their differentiation state more thoroughly and comprehensively, we profiled gene expression of control (siCtrl)- and siRab35-transfected cells by RNA sequencing at day 5, when the morphological phenotype is well pronounced (Fig. 1A). We identified 1261 differentially expressed genes (log_2_ fold change>|0.58|, *P*-adjusted<0.01) (Fig. 1B, Table S3). Interestingly, among the downregulated genes in siRab35-transfected cells, we found typical hepatocyte-associated genes, e.g. *Cyp3a11*, *Serpina1b/e, Pck1* and *Apom* (MacParland et al., 2018; Liang et al., 2022). Furthermore, KEGG Pathway enrichment analysis showed that the downregulated genes (772) were enriched in pathways typical for hepatocyte function (Fig. 1C, Table S3). In contrast, we observed significant upregulation of several genes typically not expressed in hepatocytes but related to vectorial polarity and cholangiocyte identity, e.g. *Ap1m2*, *Mal*, *Cav1*, *Rab25*, *Lamb3*, *Grhl2* and *Tacstd2* (Fig. 1B, Table S1) (Senga et al., 2012; Yamada et al., 2020; Ramnarayanan et al., 2007; Folsch, 2015; Segal et al., 2019; Aizarani et al., 2019).

To determine which of the upregulated genes following Rab35 knockdown are associated with cholangiocyte fate, we compared our data with published RNA-sequencing data of cholangiocytes and hepatocytes isolated from mouse embryonic livers at E17.5 (Yang et al., 2023). We found that 81 of the upregulated genes are enriched in cholangiocytes compared to hepatocytes (Table S3). Among these were the transcription factors (TFs) *Lin28a*, *Ghrl1* and *Ghrl2*, and known Grhl2-target genes, e.g. *Rab25*, *Cldn3*, *Tjp3* and *Sema3b* (Senga et al., 2012; Hinze et al., 2018; Varma et al., 2012).

To verify that changes in gene expression upon Rab35 knockdown were also reflected at the protein level, we performed proteomic analysis at day 6. Consistent with the RNA-sequencing results, Rab35 knockdown reduced the expression of several hepatocyte-associated proteins, e.g. cytochrome P450 3A11 (Cyp3a11), alpha-1-antitrypsin 1-2 (Serpina1b) and apolipoprotein M (Apom). Conversely, it increased the expression of vectorial polarity- and cholangiocyte identity-associated proteins, e.g. caveolin 1 (Cav1), AP-1 complex subunit mu-2 (Ap1m2) and laminin β3 (Lamb3) (Fig. 1D). Importantly, immunofluorescence microscopy revealed Tacstd2 (also known as Trop2) and caveolin 1 expression specifically in the cells forming cysts (Fig. 1E, Fig. S1C). Similar to Rab35 silencing, knockdown of Dennd1b or Acap2 decreased *Cyp3a11* and *Pck1* mRNA levels, but increased *Ap1m2*, *Cav1*, *Grhl2* and *Tacstd2* expression, and induced Tacstd2 expression in cysts (Fig. 1F, Fig. S1D).

During hepatoblast isolation, a small number of mesenchymal cells are co-isolated (Fig. S1E). Portal mesenchyme contributes to hepatoblast biliary fate specification via Notch signalling (Zong et al., 2009; Hofmann et al., 2010), raising the possibility that cell–cell contacts between mesenchymal cells and hepatoblasts may be responsible for the upregulation of cholangiocyte-associated genes upon Rab35 silencing. However, the observation that the majority of Tacstd2-positive hepatoblasts in the cysts were not in contact with mesenchymal cells (Fig. S1E) argues against this possibility.

These findings suggest that, in addition to causing a switch from multi-axial hepatocyte polarity to vectorial epithelial polarity (similar to bile duct cells), the silencing of Rab35, Dennd1b and Acap2 impairs hepatoblast-to-hepatocyte differentiation and increases the expression of cholangiocyte-associated genes.

### Isotropic expansion of apical lumina precedes transcriptional changes upon Rab35 silencing

**Fig. 2.**
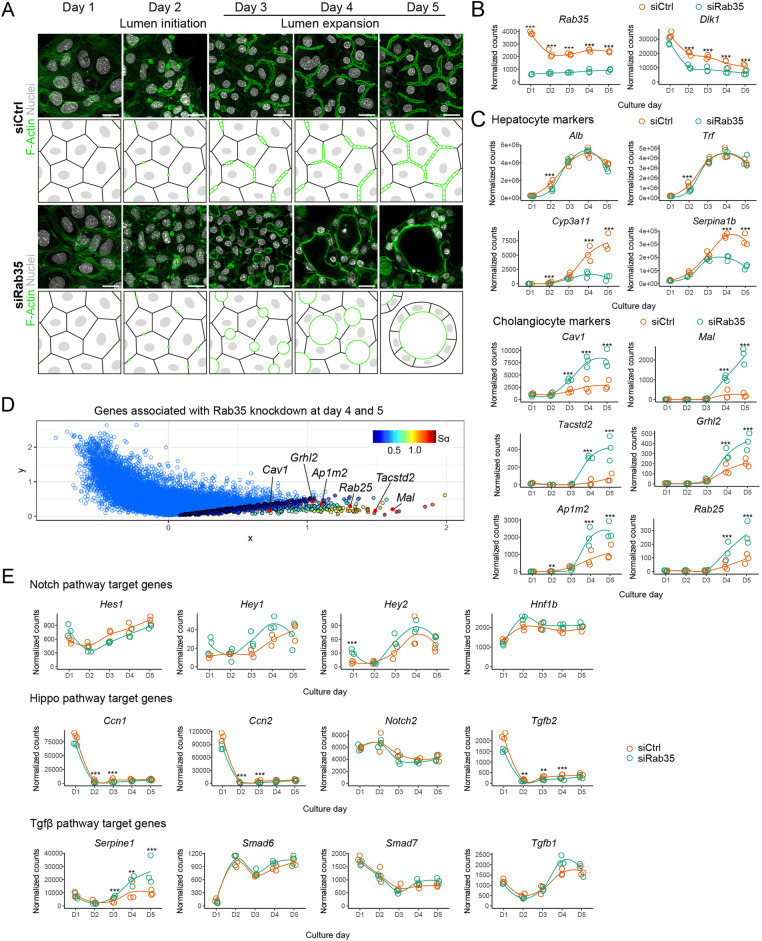
**Isotropic lumen expansion precedes transcriptional changes following Rab35 silencing.** (A) Fluorescence microscopy and schematics showing *in vitro* hepatoblast-to-hepatocyte differentiation upon siCtrl- and siRab35-transfection. Green lines in schematics indicate the subapical actomyosin cortex. Scale bars: 20 µm. (B,C) Expression profiles of *Rab35* and *Dlk1* (B) and hepatocyte markers and vectorial polarity- and cholangiocyte identity-associated genes (C) in siCtrl- and siRab35-transfected cells (*n*=3). (D) Association plot for the cluster of siRab35 day 4 and 5 samples. S_α_ of a gene is a ranking score of its cluster specificity. (E) Expression profiles of Notch, Hippo and Tgfβ targets in siCtrl- and siRab35-transfected cells. **P-adjusted<0.01; ***P-adjusted<0.001. See Materials and Methods for statistical analyses. D, day of culture.

The effects of silencing Rab35 or its partners on both the establishment of hepatocyte polarity and hepatoblast-to-hepatocyte differentiation, raised the question of whether these two processes are linked. Rab35 is a regulator of cell polarity via its role in actin organization and endocytic recycling (Klinkert and Echard, 2016). Rab35 silencing may affect the recycling of cell surface receptors involved in signalling hepatoblast cell fate decisions, resulting in the expression of cholangiocyte-related genes that lead to the formation of cyst-like lumina instead of BC. Alternatively, isotropic expansion of the apical lumen and/or subsequent changes in cell polarity phenotype could modulate a mechanotransduction pathway that feeds back on the cell transcriptional programme (Meyer et al., 2020; Kaylan et al., 2018). To distinguish between these scenarios, we examined the temporal dynamics of gene expression changes in relation to lumen formation throughout the 5-day culture of siCtrl- and siRab35-transfected cells (Fig. 2A, Table S4). We first confirmed hepatocyte differentiation in siCtrl-transfected cells. Expression of the hepatoblast marker *Dlk1* decreased over time, whereas the expression of hepatocyte markers, e.g. *Alb*, *Trf*, *Cyp3a11*, and *Serpina1b*, increased, reflecting the differentiation of hepatoblasts into foetal hepatocytes *in vivo* (Fig. 2B,C, Fig. S2A,B). Although Rab35 knockdown did not affect the expression of some hepatocyte markers (*Alb*, *Trf*), it clearly decreased the expression of others (*Cyp3a11*, *Serpina1b*), on days 4 and 5 (Fig. 2C, Fig. S2B)

To determine the timing of gene expression divergence between siCtrl- and siRab35-transfected cells, we visualized the complete timecourse dataset using a correspondence analysis 3D biplot (File S1). Individual replicates of the samples clustered together, and the samples separated according to the culture timeline. siCtrl and siRab35 samples were close to each other in the 3D plot on days 1-3, indicating similar global gene expression patterns during these days. Bifurcation between the siCtrl- and siRab35-transfected cells was observed at day 4, with notable differences in gene expression. Using an association plot (Fig. 2D), a two-dimensional plot that depicts genes associated with a cluster of samples (Gralinska and Vingron, 2023), we identified 588 genes characteristic for the siRab35-transfected samples at days 4 and 5 (Table S5). Among those, we confirmed the upregulation of vectorial polarity- and cholangiocyte identity-associated genes, including *Ap1m2*, *Mal*, *Cav1*, *Rab25*, *Tacstd2* and the TF *Grhl2* (Fig. 2D). Hence, the downregulation of hepatocyte-associated genes and upregulation of cholangiocyte-associated genes occurred simultaneously from day 4 onwards, i.e. one day after differences in lumen growth (anisotropic versus isotropic expansion) emerge between siCtrl- and siRab35-transfected cells (Fig. 2A).

Previous work has demonstrated the role of Notch, Hippo and Tgfβ signalling in hepatoblast specification towards a cholangiocyte fate (Zong et al., 2009; Clotman et al., 2005; Yimlamai et al., 2014). To determine whether these signalling pathways may account for the expression of cholangiocyte and vectorial polarity-associated genes upon Rab35 knockdown, we plotted the expression patterns of prototypical Notch, Hippo and Tgfβ target genes (Fig. 2E). None of the selected Notch and Hippo targets was upregulated following Rab35 knockdown and, except for *Serpine1*, Tgfβ targets were also not elevated, suggesting that modulation of canonical Notch, Hippo, or Tgfβ signalling is not the mechanism whereby Rab35 knockdown affects hepatocyte differentiation.

We noted that the expression of various cholangiocyte-associated genes was slightly increased in siCtrl-transfected cells at days 4 and 5 (e.g. Fig. 2C, *Grhl2*, *Ap1m2*, *Tacstd2*). Indeed, at day 5 we occasionally observed Tacstd2-positive cells in control wells. Interestingly, Tacstd2 expression correlated with hepatocytes facing abnormally large apical lumina, typically found in regions with high cell density at the rims of the wells (Fig. S3). These observations further strengthen the correlation between a more spherical apical lumen and cholangiocyte gene expression.

In conclusion, we observed that the loss of apical bulkheads and consequent isotropic apical lumen expansion precede the transcriptional changes upon Rab35 silencing. This observation alone does not prove that Rab35 knockdown influences gene expression through its effect on lumen morphology. Modulation of lumen morphology and gene expression could occur with different kinetics via independent mechanisms. Nevertheless, the sporadic expression of Tacstd2 in control cells with enlarged apical lumina suggests that changes in apical lumen morphology (i.e. enlarged, more spherical, lumina instead of tubular BC) correlate with the gene expression profile of differentiating hepatoblasts, independent of Rab35 modulation.

### Luminal pressure and subapical actomyosin contractility influence hepatocyte differentiation

We next tested whether apical lumen morphology is sufficient to influence hepatocyte differentiation. We set to perturb lumen morphology by reducing the contractility of the subapical actomyosin cortex and by increasing luminal pressure. We then assessed the effect of these perturbations on the gene expression profile by RNA sequencing.

**Fig. 3.**
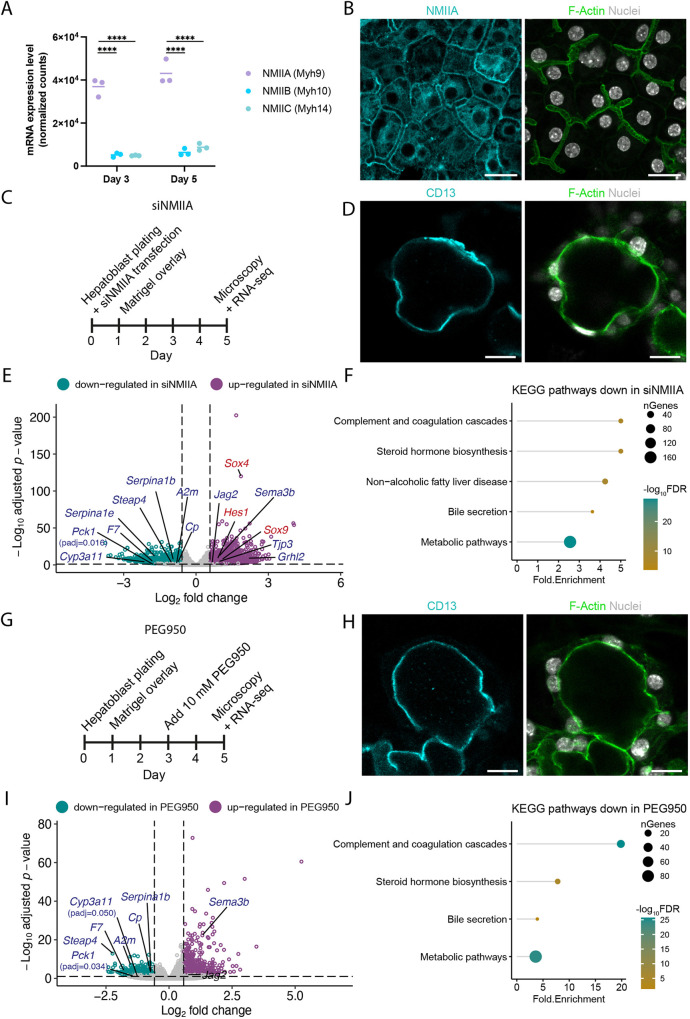
**Modulation of actomyosin contractility and luminal pressure impairs hepatocyte differentiation.** (A) Expression levels of non-muscle myosin (NMII) isoforms in siCtrl-transfected hepatoblasts at days 3 and 5 (*n*=3). ****P<0.0001. (B) Differentiating hepatoblasts at day 5. (C) Timeline of the NMIIA knockdown experiment. (D) siNMIIA-transfected differentiating hepatoblasts. (E) Differentially regulated genes upon siNMIIA-transfection, compared to siCtrl (*n*=4). Bona fide cholangiocyte markers are highlighted in red. (F) Selected pathways from KEGG pathway enrichment analysis based on genes downregulated in siNMIIA-transfected cells. (G) Timeline of the PEG950 treatment protocol. (H) PEG950-treated differentiating hepatoblasts. (I) Differentially regulated genes upon PEG950 treatment (*n*=4) (J) Selected pathways from KEGG pathway enrichment analysis based on genes downregulated in PEG950-treated cells. See Materials and Methods for statistical analyses. Scale bars: 20 µm.

To reduce the contractility of the subapical cortex, we silenced non-muscle myosin IIa (NMIIA, *Myh9*), which is the most abundant NMII isoform in differentiating hepatoblasts and localizes to the subapical actomyosin cortex (Fig. 3A,B, Fig. S4A). NMIIA knockdown caused effects similar to Rab35 silencing: (1) isotropic apical lumen expansion, and the reorganization of hepatocytes into cysts with vectorial polarity (Fig. 3C,D, Fig. S4B); (2) reduced expression of hepatocyte markers, e.g. *Cyp3a11* and *Serpina1b*, and downregulation of genes associated with hepatocyte functions (Fig. 3E,F, Table S6); and (3) increased expression of 32 out of the 81 genes that are upregulated upon Rab35 knockdown and enriched in cholangiocytes at E17.5 *in vivo* (Fig. 3E, Table S6). However, although partially overlapping, the changes in gene expression upon NMIIA and Rab35 knockdown were not completely identical. For example, NMIIA silencing did not significantly increase the expression of *Tacstd2* and *Cav1*, but did increase the expression of bona fide cholangiocyte markers, e.g. *Sox4/9* and *Hes1.*

Second, we raised luminal pressure of nascent BC by treating the cells with polyethylene glycol 950 (PEG950). This osmotically active biologically inert polymer is transported into the BC lumen (Javitt, 2014), resulting in water influx into the BC and elevated hydrostatic pressure. PEG950 treatment between days 3 and 5 severely dilated the BC and even induced the formation of cysts with vectorial polarity (Fig. 3G,H, Fig. S4C). PEG950 treatment also downregulated hepatocyte markers and pathways typical for hepatocyte function (Fig. 3I,J, Table S6). The effect of PEG950 on cholangiocyte-associated genes was less pronounced, upregulating eight out of the 81 genes that are upregulated upon Rab35 knockdown and enriched in E17.5 cholangiocytes (Table S6). This more moderate effect may be due to the shorter duration of the treatment compared to Rab35 and NMIIA knockdown.

Altogether, these findings demonstrate that the induction of isotropic apical lumen expansion, at the expense of tubular BC elongation, impairs hepatoblast-to-hepatocyte differentiation. Furthermore, the upregulation of cholangiocyte-associated genes in response to Rab35 and NMIIA knockdown, as well as the sporadic Tacstd2 expression in control cells facing abnormally large BC, suggest a link between isotropic lumen expansion and hepatoblast-to-cholangiocyte differentiation. Clearly, we need to uncover the molecular mechanism(s) by which isotropic expansion of the apical lumen influences hepatoblast differentiation. We favour the existence of mechanosensing and mechanotransduction pathways that could mediate the transcriptional changes upon loss of lumen anisotropy. The Notch and Hippo signalling pathways are sensitive to mechanical cues and have been implicated in cholangiocyte specification (Dupont et al., 2011; Gordon et al., 2015; Hunter et al., 2019; Zong et al., 2009; Yimlamai et al., 2014). However, the absence of transcriptional activation of canonical Notch and Hippo target genes (Fig. 2E) do not support their involvement.

To identify gene regulatory networks that may be modulated in response to changes in apical lumen morphology, we compared TFs that are differentially regulated in response to Rab35 knockdown, NMIIA knockdown, and PEG950 treatment. We identified three hepatocyte-enriched TFs – Bhlha15 (also known as Mist1), Klf12 and Nr1i3 – that were downregulated in all three conditions. Interestingly, Bhlha15 and Nr1i3 have previously been linked to Cyp3a11 expression (Wei et al., 2002; Chikada et al., 2015). Conversely, the cholangiocyte-enriched TFs Grhl1/2 and Lin28a, as well as several Grhl2 targets, were upregulated following Rab35 and NMIIA knockdown. These TFs are thus candidate players in the transcriptional response to changes in apical lumen morphology during liver development.

### The interplay of cell polarity and cell fate decisions in the developing liver

**Fig. 4.**
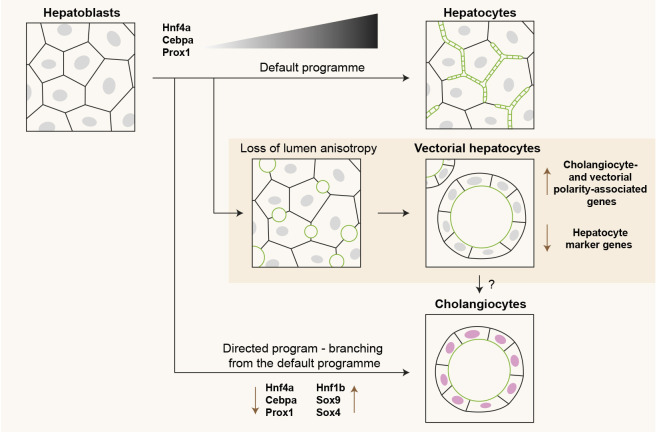
**The consequence of a loss in lumen anisotropy on hepatoblast differentiation.** Hepatoblasts differentiate via default and directed pathways into hepatocytes and cholangiocytes, respectively. Here, we demonstrate that the loss of lumen anisotropy, and the acquisition of vectorial polarity, impairs the ongoing default differentiation programme. This results in the formation of vectorially polarized cells with a mixed hepatocyte-cholangiocyte gene expression profile, which we termed vectorial hepatocytes. Green represents subapical actomyosin cortex. Pink represents nuclei containing Sox9.

During liver development, most hepatoblasts differentiate into hepatocytes through a default programme mediated by progressively strengthened gene regulatory networks of hepatocyte TFs (Yang et al., 2017; Kyrmizi et al., 2006; Yang et al., 2023). A small proportion of hepatoblasts receives signals from the portal mesenchyme to take on a cholangiocyte fate and form bile ducts. This process involves several signalling pathways, e.g. Notch, Hippo and Tgfβ, which drive the expression of cholangiocyte-associated TFs, e.g. Sox9 and Hnf1β (Zong et al., 2009; Clotman et al., 2005; Yimlamai et al., 2014; Lee et al., 2016; Wu et al., 2017). Here, we found an additional level of cell fate regulation that integrates control of gene expression with cell polarity and lumen morphogenesis, adding to the body of evidence that the establishment of epithelial polarity is not merely an endpoint to cell differentiation, but actively contributes to cell fate decisions (Motegi et al., 2020). We showed that the failure to establish the typical elongated BC results in the generation of cysts, in which the cells assume a cholangiocyte-like vectorial polarity, have reduced expression of hepatocyte-specific genes and have increased expression of cholangiocyte-associated genes (Fig. 4).

*In vivo*, bile ducts develop through a stage of transient asymmetry whereby one side is composed of Sox9-positive cholangiocytes and the other of HNF4α-positive hepatoblasts (Antoniou et al., 2009). These hepatoblasts acquire vectorial polarity, contribute to an expanding multicellular lumen, and eventually differentiate into cholangiocytes as the bile duct matures. Based on our results, it will be interesting to investigate whether and how the acquisition of vectorial polarity is an additional regulatory step in the differentiation of hepatoblasts towards cholangiocytes in asymmetric bile ducts. Addressing this question requires the biophysical and molecular mechanisms that link cell organization and cell fate to be elucidated. Additionally, apical lumen morphology and hepatocyte polarization may influence cell fate in the context of liver disease. Cholestatic liver diseases are often associated with the appearance of so-called hepatocyte rosettes. In these structures, hepatocytes are vectorially polarized towards a common enlarged apical lumen, and some cells do express cholangiocyte markers, such as Sox9 (Lin et al., 2022; Mayer et al., 2023). The formation of hepatocyte rosettes suggests that the feedback of cell polarity and lumen formation on cell (de)differentiation described here may operate under pathological conditions.

In conclusion, our data demonstrate that anisotropic BC elongation is required for hepatoblast-to-hepatocyte differentiation. Further studies into the mechanisms that link cell polarity, morphogenesis, and cell fate decisions in the liver are highly relevant to understanding how liver tissue can be resilient to changes in luminal pressure during development and disease.

## MATERIALS AND METHODS

### Hepatoblast isolation and *in vitro* differentiation toward hepatocytes

Hepatoblast isolation from embryonic livers (E13.5, male and female embryos) of C57BL/6JOlaHsd mice and subsequent *in vitro* differentiation toward hepatocytes was performed as described by Belicova et al. (2021). In brief, embryonic livers were fragmented in Liver Perfusion Medium (Thermo Fisher Scientific; 17701-038) and subsequently digested in Liver Digest Medium (Thermo Fisher Scientific; 17703-034) supplemented with 10 µg/ml DNase I (Sigma-Aldrich; DN25). Erythrocytes were lysed in red blood cell lysis buffer: 155 mM NH_4_Cl, 10 mM KHCO_3_, and 0.1 mM EDTA, pH 7.4. Next, the cells were blocked with anti-mouse CD16/CD32 blocking antibody (BD Biosciences; 553142; rat) and Dlk1^+^ hepatoblasts were labelled with anti-Dlk mAb-FITC (MBL; D187-4; rat) and anti-FITC MicroBeads (Miltenyi Biotec; 130–048-701). Labelled Dlk^+^ hepatoblasts were isolated using a magnetic column (Miltenyi Biotec; 130-024-201) according to the manufacturer's protocol. Isolated hepatoblasts were seeded in 96-well cell culture microplates (Greiner; 655090) coated with Matrigel (Corning; 356231) at a density of 13,000 cells/well in expansion media: DMEM/F-12, GlutaMAX supplement (Thermo Fisher Scientific; 31331028), 10% fetal bovine serum, 1× insulin-transferrin-selenium-ethanolamine (Gibco; 51500–056), 0.1 µM dexamethasone (Sigma-Aldrich; D1756), 10 mM nicotinamide (Sigma-Aldrich; N0636), 10 ng/ml human hepatocyte growth factor (in-house production), and 10 ng/ml mouse epidermal growth factor (in-house production). After 24 h, the cells were overlaid with differentiation media: MCDB131 (Thermo Fisher Scientific; 10372019), 2 mM L-glutamine (Thermo Fisher Scientific; M11-004) 0.25× insulin-transferrin-selenium-ethanolamine, and 0.1 µM dexamethasone, containing Matrigel to a final concentration of 5% vol/vol. Cells were cultured in a humidified incubator with 5% CO_2_ at 37°C for a maximum of 6 days. For the PEG950 treatment, the culture medium of the cells was replaced at day 3 with differentiation medium containing 10 mM PEG950 (Merck; P3515).

Embryonic livers were obtained in accordance with German animal welfare legislation in the animal facility of the Max Planck Institute of Molecular Cell Biology and Genetics (MPI-CBG), Dresden, Germany. Protocols were approved by the Institutional Animal Welfare Officer (Tierschutzbeauftragter), and necessary licenses were obtained from the regional Ethical Commission for Animal Experimentation of Dresden, Germany (Tierversuchskommission, Landesdirektion Dresden).

### siRNA-mediated knockdown

Cells were reverse-transfected with siRNAs upon plating using Lipofectamine RNAiMAX (Thermo Fisher Scientific; 13778075) according to the manufacturer's protocol. The final siRNA concentration per well was 10 nM (siRab35, siNMIIA) or 20 nM (siAcap2, siDennd1b) at a concentration of 0.1 vol/vol % Lipofectamine RNAiMAX. siLuciferase was used as control for siAcap2, siDennd1b and siNMIIA. A scrambled Rab35 siRNA (scrRab35) was used as control for siRab35 in all experiments, except for the RNA-sequencing experiments shown in Figs 1 and 3, for which siLuciferase was used as control. siRNAs used were: siLuciferase (sense 5′-cuuAcGcuGAGuAcuucGAdTsdT-3′, antisense 5′-UCGAAGuACUcAGCGuAAGdTsdT-3′), siNMIIA (Myh9) (Thermo Fisher Scientific; MSS237342), siRab35 (sense: 5′-CGcAAGGAGGAGcAUUUuATsT-3′, antisense: 5′-uAAAAUGCUCCUCCUUGCGTsT-3′), scrRab35 (sense: 5′- GuAcGuAAucGGcAGAAGuTsT-3′, antisense: 5′-ACUUCUGCCGAUuACGuACTsT-3′), siDennd1b (sense: 5′-AuGAGAGGcGcAucAuuAuTsT–3′, antisense: 5′-AuAAUGAUGCGCCUCUcAUTsT-3′) and siAcap2 (sense: 5′-cAAuGuGcuucAGucAAAATsT-3′, antisense: 5′-UUUUGACUGAAGcAcAUUGTsT-3′).

### Immunofluorescence staining and confocal microscopy

Cells were fixed with 3% paraformaldehyde in PBS for 20 min at room temperature (RT), washed with PBS, and blocked using blocking buffer (2% bovine serum albumin and 0.05% saponin in PBS) for 1 h at RT. Cells were incubated with primary antibodies diluted in blocking buffer at 4°C overnight. Primary antibodies used were: anti-CD13 (NB100-64843; rat; Novus Biologicals; 1:500), anti-NMIIA (GTX113236; rabbit; GeneTex; 1:100), anti-Desmin (AF3844; goat; R&D Systems; 1:200), anti-Trop2 (ab214488; rabbit; Abcam; 1:200) and anti-Caveolin (D46G3; rabbit; Cell Signaling Technology; 1:200). Phalloidin-488 (A12379; Thermo Fisher Scientific) and DAPI were included in the primary antibody solutions. Following washing with PBS, cells were incubated with secondary antibodies diluted in blocking buffer for 2 h at RT. Secondary antibodies used were: goat anti-rabbit Alexa Fluor 647 (A21244; Thermo Fisher Scientific; 1:500), goat anti-rat Alexa Fluor 647 (A21247; Thermo Fisher Scientific; 1:500), donkey anti-rabbit Alexa Fluor 647 (A31573; Thermo Fisher Scientific; 1:500) and donkey anti-goat Alexa Fluor 568 (A11057; Thermo Fisher Scientific; 1:500). An LSM700 inverted laser scanning confocal microscope (Zeiss) equipped with a 40×/1.2 C-Apochromat, water, DIC objective (Zeiss), and Zen imaging software (Zeiss) were used for confocal microscopy. Fiji (Schindelin et al., 2012) was used for image analysis.

### RNA isolation and RT-quantitative PCR (qPCR)

RNA was isolated using the RNeasy Mini Kit (QIAGEN; 74104) including DNase I (QIAGEN; 79254) treatment. Cells were lysed with RNeasy lysis buffer supplemented with 40 mM DTT. cDNA was synthesized using the ProtoScriptII First Strand cDNA Synthesis Kit (NEB; E6560S), following the manufacturer's protocol with the Random Primer Mix and the RNA denaturation step. qPCR was performed with a Roche LightCycler 96 using FastStart Essential DNA Green Master (Roche; 06402712001). As reference gene, we used the housekeeping gene *Rplp0*. Primer sequences used are described in Table S2. Normalized relative gene expression values and percentage knock down were calculated using the ΔΔCq method. GraphPad Prism version 9.0 (GraphPad software) was used for data analysis and visualization.

### RNA sequencing

For the RNA-sequencing experiment in Fig. 1B, samples were collected from three biological replicates of *in vitro* cultured E13.5 Dlk^+^ hepatoblasts transfected with siRab35, or control siRNA (siLuciferase) at day 5 of the culture. For the timecourse RNA sequencing in Fig. 2, samples were collected every day from day 1 to day 5 from scrRab35- and siRab35-transfected *in vitro*-cultured E13.5 Dlk^+^ hepatoblasts in three biological replicates. Rab35 mRNA knockdown was verified by RT-qPCR. Importantly, even though different control siRNAs were used (siLuciferase versus scrRab35), the results of the RNA-sequencing experiment on day 5 (Fig. 1B) were comparable to the results of the timecourse RNA-sequencing experiment on day 5 (Fig. S5A,B), indicating that the results were reproducible. For the RNA-sequencing experiments in Fig. 3, samples were collected from four biological replicates of *in vitro*-cultured E13.5 Dlk^+^ hepatoblasts transfected with siRNA targeting NMIIA (siMyh9), or control siRNA (siLuciferase) at day 5 of the culture, and from hepatoblasts treated with PEG950-containing culture medium or control medium. The integrity of RNA was measured using an Agilent 2100 Bioanalyzer. Preferentially, only samples with RNA integrity number (RIN) >9.0 were used. mRNA was isolated from the total RNA by poly-dT enrichment using the NEBNext Poly(A) mRNA Magnetic Isolation Module according to the manufacturer's instructions, fragmented to 350 nt, and libraries were prepared using the NEBNext UltraTM II Directional RNA Library Prep Kit for Illumina. For Illumina flowcell production, samples were equimolarly pooled and distributed on all lanes used for 75 bp single-read sequencing on an Illumina NextSeq 500 (Fig. 1B) or 100 bp paired-end sequencing on an Illumina NovaSeq 6000 (Figs 2 and 3), resulting in an average of 33 Mio (Fig. 1B), 67 Mio (Fig. 2), 74 Mio (Fig. 3) sequenced fragments per sample.

Analysis of RNA-sequencing data (including the published RNA-sequencing data of cholangiocytes and hepatocytes isolated from mouse embryonic livers from Yang et al., 2023; GSE142089) was performed in R (v.4.1.0) (https://www.R-project.org/) using following packages: DESeq2 (v.1.40.0) (Love et al., 2014), ggplot2 (v.3.4.4) (https://ggplot2.tidyverse.org), pheatmap (v.1.0.12) (https://CRAN.R-project.org/package=pheatmap), EnhancedVolcano (v.1.18.0) (https://bioconductor.org/packages/EnhancedVolcano), VennDiagram (v.1.7.0) (https://CRAN.R-project.org/package=VennDiagram), dplyr (v.1.1.2) (https://CRAN.R-project.org/package=dplyr), ggpubr (v.0.6.0) (https://CRAN.R-project.org/package=ggpubr), cowplot (v.1.1.1) (https://CRAN.R-project.org/package=cowplot). The online application ShinyGo (v.0.80) (Ge et al., 2020) was used for gene ontology enrichment analysis (KEGG pathways database). DESeq2 package was used with the default parameters (e.g. Benjamini–Hochberg correction for *P*-value). The replicates in all experiments clustered well together and batch correction was not required. Genes with a log2 fold change>|0.58|and an adjusted *P*-value <0.01 were considered to be differentially expressed. Correspondence analysis (CA) of the timecourse RNA-sequencing data was conducted using the Bioconductor package APL (v.0.99.5) (Gralinska et al., 2022). The function ‘cacomp’ was applied to the matrix of normalized counts (output of the DESeq2 package) using the default parameters, except for the top set to 19,331. The interactive 3D plot was generated using Plotly (Plotly Technologies Inc.) (Sievert, 2020). The association plot for the cluster of Rab35 siRNA-treated samples from days 4 and 5 was generated using the first five correspondence analysis dimensions. Cluster-specificity scores S_α_ were computed based on 500 permutations (parameter reps in function apl_score).

### Proteomics

On day 6 of the hepatoblast culture, cells were washed twice with PBS at RT, placed on ice and washed three times for 10 min each wash with cell recovery solution (Corning; CLS354270) to remove Matrigel. Cells were lysed at RT for 10 min using freshly prepared lysis buffer: RIPA buffer with EDTA and EGTA (Alfa Aesar/Thermo Fisher Scientific)+5% SDS+2× cOmplete protease inhibitor cocktail (Roche). After centrifugation at 14,000 rpm (16,900 ***g***) for 10 min at RT, the supernatant was used for proteomics. Label-free protein quantification was performed with four biological replicates per condition, prepared and measured in a block-randomized fashion (Burger et al., 2021). After bead beating with a TissueLyser II (QIAGEN) and 0.5 mm stainless steel beads (Next Advance) for two rounds of 5 min each at 25 Hz at 4°C to shear DNA/RNA, samples were centrifuged at 13,000 ***g*** for 10 min at RT. Proteins from the supernatants were precipitated in 85% acetone and 30 mM NaCl by incubation at RT for 30 min at 800 rpm in a shaker (Nickerson and Doucette, 2020). Protein pellets were resuspended in 1× S-Trap lysis buffer [5% SDS, 50 mM TEAB (Merck), pH 8.5] and 50 µg of the sample protein was digested using the S-trap micro spin column digestion protocol from ProtiFi LLC. Washes were performed six times with 200 µl each and digestion was performed with 0.75 µg Trypsin/Lys-C Mix (Promega) per 10 µg total protein. Eluted peptides were dried in a RVC 2-25 rotary vacuum concentrator (Martin Christ Gefriertrocknungsanlagen GmbH), and stored at −20°C. Before measurement, samples were resuspended in 0.2% formic acid (Merck). Total peptide concentration was adjusted to 0.15 µg/µl according to the absorbance at 280 nm against a Mass Spec-Compatible Human Protein Extract/Digest calibration curve (Promega), using a Thermo Nanodrop 1000 ND-1000 spectrophotometer (Thermo Fisher Scientific).

The liquid chromatography system was a Thermo Dionex UltiMate 3000 UHPLC system with an Acclaim PepMap precolumn (100 µm×20 mm, C18, 5 µm, 100 Å) and a 50 cm µPAC column array (all from Thermo Fisher Scientific); 5 µl of the sample was loaded onto the precolumn at 5 µl/min and eluted with a linear gradient with two slopes at 0.5 µl/min and 0.1% formic acid. The percentage of acetonitrile (Thermo Fisher Scientific) was increased from 0% to 17.5% over two-thirds of the gradient length and from 17.5% to 35% in the last third. Both columns were then rinsed with 95% acetonitrile for 7 min. At least one blank injection with a 25-min gradient was performed between each sample injection to reduce carryover. Sample measurements with 120-min gradients were performed. We used a μPAC Flex iON interface (Pharma Fluidics/Thermo Fisher Scientific) equipped with a 5 cm nanoESI emitter with a 20 µm inner diameter (Fossiliontech). Electrospray was performed with a spray voltage of 2.5 kV, a capillary temperature of 280°C, and an S-lens RF value of 50.

Mass spectrometry data acquisition was performed using a Q Exactive HF mass spectrometer (Thermo Fisher Scientific). Data-independent acquisition included a low-resolution MS1 scan in centroid mode covering the *m/z* range of 395-971 with a resolution R*_m/z_*_=200_ of 30,000, an AGC of 3×10^6^, 40 ms injection time, and a fixed first mass at *m/z* 100. The following 32 MS2 scans covered the *m/z* range of 400 to 966 (after demultiplexing) with a total cycle time of about 3 s. MS2 spectra were acquired under normalized collision energy of 24 using an *m/z* isolation window width of 18, maximum filling time of 55 ms under AGC of 1×10^6^, and resolution R_m/z=200_ of 30,000 in centroid mode. Data-independent acquisition (DIA) acquisition was performed in a staggered manner (Amodei et al., 2019). The raw files were converted to mzML format and demultiplexed with MSConvert v.3.0.2 (Adusumilli and Mallick, 2017). Analysis against mouse reference proteome UP000000589 SwissProt canonical+isoform as of 07.02.2022 with added MaxQuants common contaminants was performed with DIA-NN v.1.8 (Demichev et al., 2020). Settings were --min-fr-mz 100 --max-fr-mz 2000 --cut K*,R* --missed-cleavages 1 --min-pep-len 7 --max-pep-len 34 --min-pr-mz 400 --max-pr-mz 966 --min-pr-charge 2 --max-pr-charge 4 --var-mods 1 --var-mod UniMod:35,15.994915,M --double-search --individual-mass-acc --individual-windows --smart-profiling --pg-level 2 --species-genes --peak-center --no-ifs-removal --no-quant-files --report-lib-info --matrix-qvalue 0.01 --nn-single-seq --fixed-mod UniMod:39,45.987721,C --strip-unknown-mods. For further analysis, the protein group matrix from DIA-NN was processed. Protein groups reported in at least two out of the total of four replicates per condition were considered as positively identified. Protein groups further characterized by a coefficient of variation below 25% were considered as quantified in that condition. Differential expression analysis of quantified protein groups was performed with Limma 3.50.0 with α=0.01 and ∣log2 fold-change∣>0.5 (Ritchie et al., 2015). Intensities plotted in Fig. 1D were obtained by label-free quantification with DIA-NN, using the default MaxLFQ normalization. DIA data acquisition and DIA-NN data processing routine were optimized and validated according to Jumel and Shevchenko (2024).

### Statistical analysis

The statistical analyses in Fig. 1D and Fig. S4A were performed using an unpaired, two-tailed *t*-test. The qRT-PCR experiments in Fig. 1F and Fig. S1B were analysed using Student's one-sample, two-tailed *t*-test against a theoretical mean of 1. The statistical analyses for Fig. 2B,C,E and Fig. S2A,B were performed in R package DESeq2 (Love et al., 2014). Statistical significance of the differences in expression levels between siCtrl- and siRab35-transfected cells (Fig. 2B,C,E, Fig. S2B) or hepatocytes and cholangiocytes (Fig. S2A) was tested for each day of the culture using Wald test with Benjamini–Hochberg method for multiple testing correction of *P*-value (pairwise comparison of two conditions). The visualization of patterns in Fig. 2B,C,E and Fig. S2A,B was aided by plotting smoothened conditional means (LOESS method). The statistical analysis in Fig. 3A was performed using an ordinary one-way ANOVA and a Tukey's multiple comparisons test with a single pooled variance. Unless specified otherwise, statistical analyses were performed using GraphPad Prism v.9.0 (GraphPad software).

## Supplementary Material



10.1242/develop.202777_sup1 Supplementary information

Table S3. Differential gene expression analysis for Rab35 knockdown. Sheet 1) Significantly up-and down-regulated genes upon Rab35 siRNA treatment compared to the control siRNA. Sheet 2) Pathways from the KEGG pathways enrichment analysis on genes down-regulated in siRab35-treated cells compared to siLuc-treated cells. Sheet 3) Size factor normalized counts for the RNA sequencing experiment of Fig. 1B. Sheet 4) Genes that are upregulated (log2 fold change > 0.58, p-adjusted value < 0.01) following Rab35 knockdown and are enriched in cholangiocytes as compared to hepatocytes at E17.5 (log2 fold change > 0.58, p-adjusted value < 0.01).

Table S4. Size factor normalized counts for the time-course RNA sequencing. Size factor normalized counts for the Ame-course RNA sequencing experiment of Fig. 2.

Table S5. Genes characteristic for the Rab35 knockdown samples at days 4 and 5. Genes characteristic for the Rab35 knockdown samples at days 4 and 5 (cluster-specificity score of a gene Sα cut-off: 0.01)

Table S6. Differential gene expression analysis for NMIIA knockdown and PEG950 treatment. Sheet 1) Significantly up-and down-regulated genes upon NMIIA siRNA treatment compared to the control siRNA. Sheet 2) Genes that are significantly upregulated upon NMIIA siRNA treatment, Rab35 siRNA treatment, and enriched in cholangiocytes at E17.5 *in vivo*. Sheet 3) Significantly up-and down-regulated genes upon PEG950 treatment compared to control. Sheet 4) Genes that are significantly upregulated upon PEG950 treatment, Rab35 siRNA treatment, and enriched in cholangiocytes at E17.5 *in vivo*.
