# Supplementary material for: Hepatocyte differentiation requires anisotropic expansion of bile canaliculi
Source: Development. 2024 Nov 21;151(22):dev202777. doi: 10.1242/dev.202777 (PMC11607689; doi:10.1242/dev.202777)
Supplement: Supplementary information [file develop-151-202777-s1.pdf]

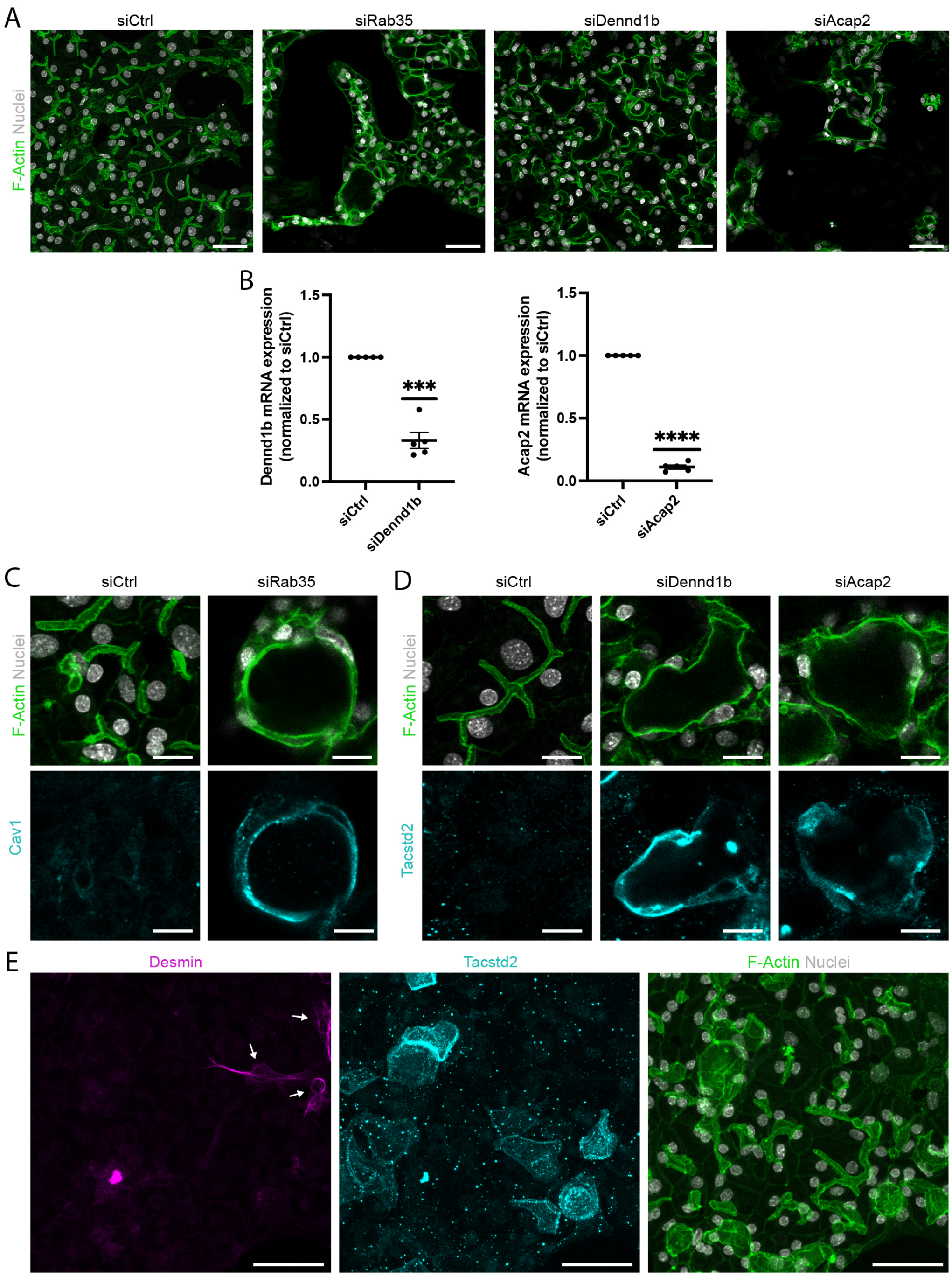

Fig. S1. Silencing of Rab35, Dennd1b, and Acap2 impairs hepatocyte polarization and differentiation. A) Larger field-of-view images associated with the cropped images in Fig. 1A. F-actin (green), nuclei (grey). Scalebars: 50 μm. B) qRT-PCR analysis showing knockdown of Dennd1b and Acap2 at day 5 of culture following transfection with Dennd1b and Acap2 siRNA, respectively (n=5). Statistical method: One-sample two-tailed t-test, theoretical mean of 1. *P<0.05; ***P<0.001; ****P<0.0001. C) Immunofluorescence microscopy of differentiating hepatoblasts transfected with control or Rab35 siRNA at day 5 of culture. Cav1 (cyan), F-actin (green) and nuclei (grey). Scalebar: 20 μm. D) Immunofluorescence microscopy of differentiating hepatoblasts transfected with control, Dennd1b or Acap2 siRNA at day 5 of culture. Cells are stained for Tacstd2 (cyan), F-actin (green) and nuclei (grey). Scalebar: 20 μm E) Immunofluorescence microscopy of differentiating hepatoblasts and desmin-positive mesenchymal cells (white arrows) transfected with Rab35 siRNA at day 5 of culture. Cells are stained for Tacstd2 (cyan), the mesenchymal marker Desmin (magenta), F-actin (green) and nuclei (grey). Scalebar: 50 μm.

Development • Supplementary information

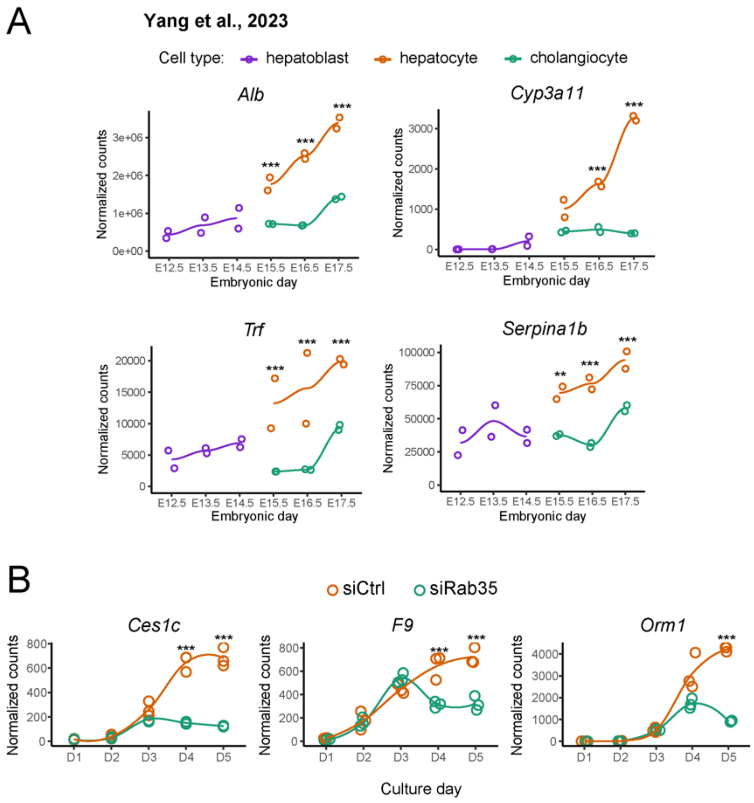

Fig. S2. Isotropic lumen expansion precedes transcriptional changes following Rab35 silencing. A) Expression profiles of hepatocyte marker genes in hepatoblasts, hepatocytes, and cholangiocytes from E12.5-E17.5 during liver development. Data obtained from (Yang et al., 2023; n=2) B) Expression profiles of hepatocyte markers in control and Rab35 siRNA (siRab35) treated samples (n=3). **, P < 0.01; ***, P < 0.001. See Methods for statistical analyses.

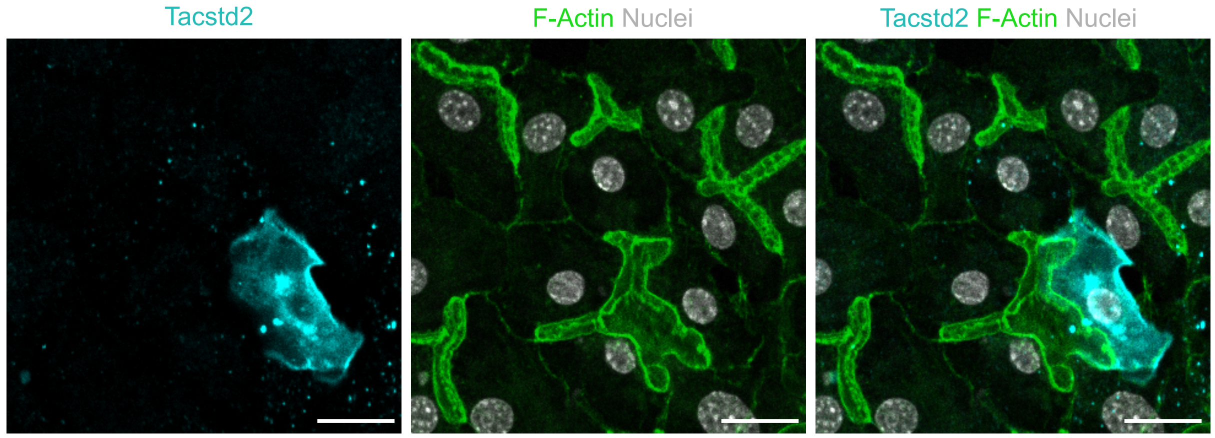

Fig. S3. Tacstd2 expression in a hepatocyte facing an abnormally large apical lumen. Immunofluorescence microscopy of differentiating hepatoblasts transfected with control siRNA (siLuciferase) at day 5 of culture. Cells are stained for Tacstd2 (cyan), F-actin (green) and nuclei (grey). Scalebar: 20 μm.

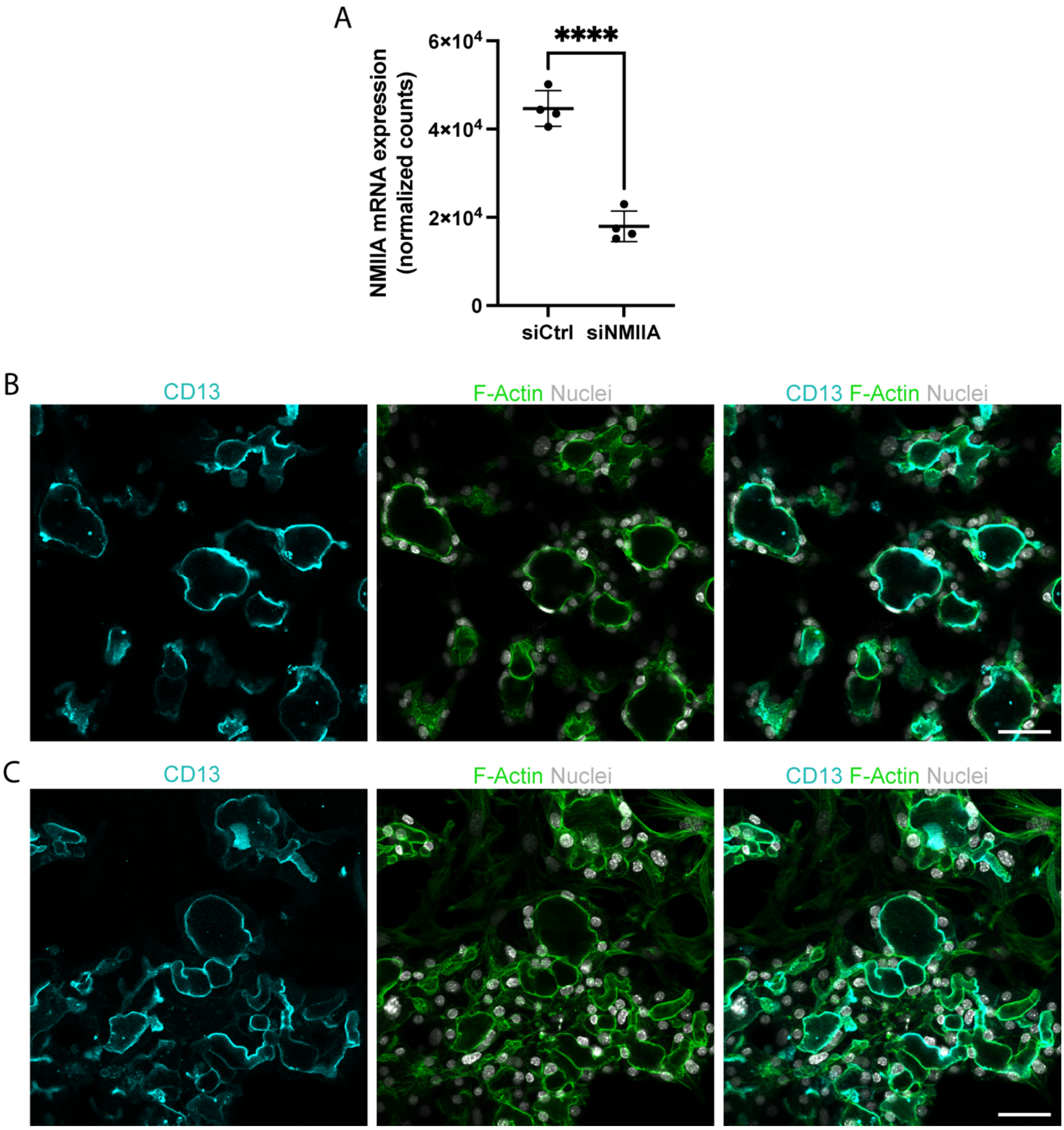

Fig. S4. Formation of hepatocyte cysts following NMIIA knockdown and treatment with PEG950 A) Normalized counts of NMIIA mRNA expression showing knockdown of NMIIA at day 5 of culture following transfection with NMIIA siRNA. N=4. Mean±SD. Statistical method: Unpaired two-tailed t-test. ****, $P < 0.0001$. B) Larger field-of-view image of siNMIIA-transfected differentiating hepatoblasts of the cropped image in Fig. 3D. CD13 (cyan), F-actin (green), nuclei (grey). Scalebar: 50 μm. C) Larger field-of-view image of PEG950-treated differentiating hepatoblasts of the cropped image in Fig. 3H. CD13 (cyan), F-actin (green), nuclei (grey). Scalebar: 50 μm.

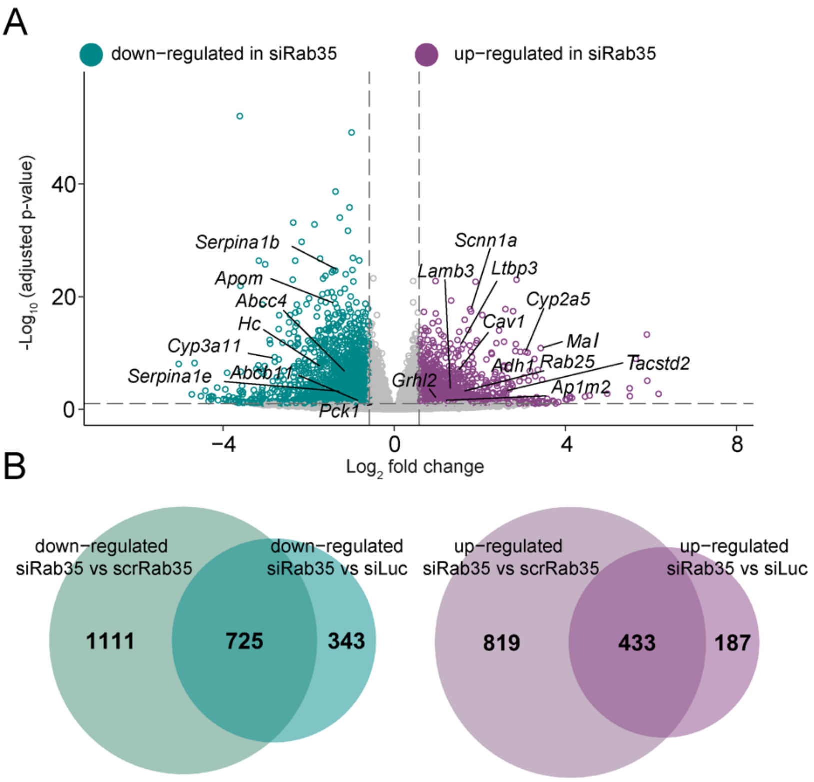

Fig. S5. Comparison of RNA-sequencing results following Rab35 silencing using siLuc and scrRab35 as control siRNAs. A) Volcano plot of results from differential gene expression analysis between control siRNA (scrRab35)- and Rab35 siRNA (siRab35)-treated cells at day 5 of the time-course experiment. Significant genes (log2 fold change > |0.58|, p-adjusted value < 0.01) in green are down-regulated in siRab35 samples and in magenta up- regulated. The labelled genes are the same as in Fig. 1B, where siRab35-transfected cells were analysed in comparison to siLuciferase-transfected cells. B) Venn diagram visualizing the number of genes identified as down- regulated or up-regulated (log2 fold change > |0.58|, p-adjusted value < 0.01) in siRab35 samples compared to control siRNA at day 5 in the experiment from Fig. 1B with siLuciferase (siLuc) as control siRNA and from the time-course experiment in Fig. 2 with scrRab35 as control siRNA, and their overlap.

Table S1. Functions and relevance of the upregulated genes following Rab35 silencing.

**Gene name**	**Protein name**	**Function and relevance to vectorial cell polarity or cholangiocyte identity**	**Reference**
Ap1m2	AP-1 complex subunit mu-2 (Ap1m2)	Ap1m2 is a component of the epithelial cell-specific clathrin adapter complex AP-1B, which is involved in polarized sorting of cargos to the basolateral membrane. Ap1m2 is selectively expressed in polarized epithelial cells, including cholangiocytes, but not expressed in hepatocytes.	(Folsch et al., 2015)
Mal	Myelin and lymphocyte protein (Mal).	Mal contributes to the direct delivery of newly synthesized single transmembrane domain and glycophosphatidylinositol-anchored proteins to the apical membrane of vectorially polarized epithelia, such as cholangiocytes. Hepatocytes do not express Mal, and deliver newly synthesized single transmembrane domain and glycophosphatidylinositol-anchored proteins to the apical membrane via an indirect pathway	(Ramnarayanan et al., 2007)

Development • Supplementary information

		called transcytosis. Exogenous expression of Mal in hepatocytes redirects these newly synthesized proteins into the direct route of apical delivery	
Cav1	Caveolin-1	Caveolin-1 is a major constituent of caveolae and has been found to be expressed at higher levels in cholangiocytes as compared to hepatocytes.	(Segal et al., 2019)
Grhl2	Grainyhead-like 2 (Grhl2)	Grhl2 is a transcription factor that is involved in epithelial morphogenesis. Expression of Grhl2 in a liver progenitor cell line enhanced the expansion of multicellular lumina via Rab25 and the tight junction proteins Claudin-3 and Claudin-4. Ghrl2 is expressed in cholangiocytes but not in hepatocytes.	(Senga et al., 2012)
Rab25	Rab25	*Rab25* is a target gene of the transcription factor Grhl2. The small GTPase Rab25 has been found to mediate the expansion of multicellular lumina in response to Grhl2 expression, by regulating the localization of Claudin-4 at tight junctions. Rab25 is expressed in cholangiocytes but not in hepatocytes	(Senga et al., 2012)
Lamb3	Laminin-β3	Laminins are extracellular matrix proteins that are core components of the basement membrane. Lamb3 expression in the liver is restricted to cholangiocytes.	(Yamada et al., 2020)
Tacstd2	Tumor-associated calcium signal transducer 2 (Tacstd2)	Tacstd2 is a cell surface protein that is primarily expressed in epithelial cells. Single-cell sequencing of human liver cells revealed that Tacstd2 expression correlates with cholangiocyte fate and anti-correlates with hepatocyte fate.	(Aizarani et al., 2019)

Table S2. Primers used for RT-quantitative PCR.

**Gene**	**Fw primer 5'-3'**	**Rev primer 5'-3'**
RpLp0	AGATTCGGGATATGCTGTTGGC	TGTCAACGTCAAGCGATGG
Rab35	TGTCAACGTCAAGCGATGG	GGTCATCATTCTTATTGCCCACT
Myh9 (NMIIA)	AGAACAAACTGAGGCGTGGG	ATCGGCCCCATCTGCTTTAC
Acap2	AACGTGGAACAGACGCTGG	TTCACTGTGCAGAGCCTGAG
Dennd1b	TTCTGCAGAGCGTGCCCAAG	GCCTGCAGAACCCAAATCGC
Tacstd2	TCCGCGGTACACTATGAGGA	CCATGAACAGTGACTCGCCT
Cav1	ACGTAGACTCCGAGGGACATC	GTGTGCGCGTCATACACTTG
Ap1m2	GCTTACTGGCCTTTCAGGCA	GGTTCGGTCGTTGTCAAAGC
Ghrl2	CGATGAGCGAGAAGGCAGC	TGATCTGCTTAGACGGTGTTGG
Cyp3a11	TGAATATGAAACTTGCTCTCACTAAAA	CCTTGTCTGCTTAATTTCAGAGGT
Pck1	ATGTGTGGGCGATGACATT	AACCCGTTTTCTGGGTTGAT

Development • Supplementary information

Table S3. Differential gene expression analysis for Rab35 knockdown. Sheet 1) Significantly up-and down-regulated genes upon Rab35 siRNA treatment compared to the control siRNA. Sheet 2) Pathways from the KEGG pathways enrichment analysis on genes down-regulated in siRab35-treated cells compared to siLuc-treated cells. Sheet 3) Size factor normalized counts for the RNA sequencing experiment of Fig. 1B. Sheet 4) Genes that are upregulated (log2 fold change > 0.58, p-adjusted value < 0.01) following Rab35 knockdown and are enriched in cholangiocytes as compared to hepatocytes at E17.5 (log2 fold change > 0.58, p-adjusted value < 0.01).

Available for download at
https://journals.biologists.com/dev/article-lookup/doi/10.1242/dev.202777#supplementary-data

Table S4. Size factor normalized counts for the time-course RNA sequencing. Size factor normalized counts for the Ame-course RNA sequencing experiment of Fig. 2.

Available for download at
https://journals.biologists.com/dev/article-lookup/doi/10.1242/dev.202777#supplementary-data

Table S5. Genes characteristic for the Rab35 knockdown samples at days 4 and 5. Genes characteristic for the Rab35 knockdown samples at days 4 and 5 (cluster-specificity score of a gene Sα cut-off: 0.01)

Available for download at
https://journals.biologists.com/dev/article-lookup/doi/10.1242/dev.202777#supplementary-data

Table S6. Differential gene expression analysis for NMIIA knockdown and PEG950 treatment. Sheet 1) Significantly up-and down-regulated genes upon NMIIA siRNA treatment compared to the control siRNA. Sheet 2) Genes that are significantly upregulated upon NMIIA siRNA treatment, Rab35 siRNA treatment, and enriched in cholangiocytes at E17.5 *in vivo*. Sheet 3) Significantly up-and down-regulated genes upon PEG950 treatment compared to control. Sheet 4) Genes that are significantly upregulated upon PEG950 treatment, Rab35 siRNA treatment, and enriched in cholangiocytes at E17.5 *in vivo*.

Available for download at
https://journals.biologists.com/dev/article-lookup/doi/10.1242/dev.202777#supplementary-data

File S1. Interactive correspondence analysis 3D biplot of the complete time-course dataset.

Available for download at
https://journals.biologists.com/dev/article-lookup/doi/10.1242/dev.202777#supplementary-data

Development • Supplementary information
