# Supplementary material for: Hepatocyte differentiation requires anisotropic expansion of bile canaliculi
Source: Development. 2024 Nov 21;151(22):dev202777. doi: 10.1242/dev.202777 (PMC11607689; doi:10.1242/dev.202777)
Supplement: Supplementary file 1 [file develop-151-202777_review_history.pdf]

Hepatocyte differentiation requires anisotropic expansion of bile canaliculi

Maarten P. Bebelman, Lenka Belicova, Elzbieta Gralinska, Tobias Jumel, Aparajita Lahree, Sarah Sommer, Andrej Shevchenko, Timofei Zatsepin, Yannis Kalaidzidis, Martin Vingron and Marino Zerial
DOI: 10.1242/dev.202777

Editor: James Wells

Review timeline

Original submission:	14 February 2024
Editorial decision:	2 April 2024
First revision received:	7 August 2024
Accepted:	17 September 2024

Original submission

First decision letter

MS ID#: DEVELOP/2024/202777

MS TITLE: Hepatocyte differentiation requires anisotropic expansion of bile canaliculi

AUTHORS: Lenka Belicova, Maarten Paul Bebelman, Elzbieta Gralinska, Tobias Jumel, Aparajita Lahree, Andrej Paul Shevchenko, Timofei Zatsepin, Yannis Kalaidzidis, Martin Vingron, and Marino Zerial

I have now received all the referees' reports on the above manuscript, and have reached a decision. The referees' comments are appended below, or you can access them online: please go to BenchPress and click on the 'Manuscripts with Decisions' queue in the Author Area.

As you will see, the referees express considerable interest in your work, but have some significant criticisms and recommend a substantial revision of your manuscript before we can consider publication. If you are able to revise the manuscript along the lines suggested, which may involve further experiments, I will be happy receive a revised version of the manuscript. Your revised paper will be re-reviewed by one or more of the original referees, and acceptance of your manuscript will depend on your addressing satisfactorily the reviewers' major concerns. Please also note that Development will normally permit only one round of major revision. If it would be helpful, you are welcome to contact us to discuss your revision in greater detail. Please send us a point-by-point response indicating your plans for addressing the referees' comments, and we will look over this and provide further guidance.

Please attend to all of the reviewers' comments and ensure that you clearly highlight all changes made in the revised manuscript. Please avoid using 'Tracked changes' in Word files as these are lost in PDF conversion. I should be grateful if you would also provide a point-by-point response detailing how you have dealt with the points raised by the reviewers in the 'Response to Reviewers' box. If you do not agree with any of their criticisms or suggestions please explain clearly why this is so.

Reviewer 1

© 2024. Published by The Company of Biologists under the terms of the Creative Commons Attribution License (https://creativecommons.org/licenses/by/4.0/).

Advance summary and potential significance to field

Earlier work from the authors (Belicova et al. J Cell Biol 2021) showed that upon silencing of the GTPase Rab35, multi-axial polarity of the hepatoblasts switches towards a vectorial apico-basal polarity. This is associated with loss of apical bulkheads and isotropic development of canaliculi which form cysts instead of tubes. It could not firmly be concluded from this work if loss of bulkheads perturbs the polarity or if perturbed polarity causes loss of bulkheads and cyst formation. The paper was subtly ambiguous about this issue and did not identify the primum movens, but nicely uncovered the existence of bulkheads. Differentiation of the hepatoblasts was not studied in depth.

Here the authors further investigate the consequences of Rab35 silencing in primary cultures of purified mouse hepatoblasts, and focus on the link between polarity and differentiation of the cells. Inhibition of Rab35 reduced expression of hepatocyte differentiation genes while stimulating the expression of markers of cholangiocyte differentiation and vectorial polarity. Changes in differentiation were preceded by isotropic expansion of the canaliculi, suggesting that differentiation is modulated by the apical morphology, not the opposite. This was further confirmed by relaxing subapical contractility (knockdown of non-muscle myosin IIa) or increasing intraluminal pressure with polyethylene glycol, both of which cause canalicular expansion and downregulation of hepatocyte genes. The authors conclude that cell polarity and lumen morphogenesis feedback to hepatocyte differentiation. Such feedback is conceptually novel in the field of liver development.

Comments for the author

The paper is clearly written and very nicely illustrated. The experiments are well designed and the conclusions are in general well supported by the data. The major weakness is in the lack of in vivo data, but the authors appropriately discuss the limitations of their experimental system.

The authors should address the following issues:

Figure 3 shows the expression of genes following knockdown of non-muscle myosin IIa and polyethylene glycol treatment. The number of tested genes is really low to support the conclusions. The authors should at least measure the 8 downregulated and 10 upregulated genes highlighted in Fig. 1B.

Point of discussion. Could the delay between morphological changes (cyst formation) and transcriptional changes following knockdown of Rab35 result from the fact that polarity changes occur faster than transcriptional changes, with polarity being - for instance - dependent on fast and direct changes in protein activity induced by Rab35 knockdown, and transcriptional changes being secondary to slower processes requiring Rab35-dependent changes in transcription factor activity and slow control of transcription ? In that case, there would be no need to consider that morphogenesis is the inducer of transcriptional changes; both morphogenesis and differentiation would be dependent on Rab35, the one being faster than the other.

Point of discussion. The fact that transcription factors of the core cholangiocyte and hepatocyte gene regulatory networks (SOX9, SOX4, HNF1b, HNF4, HNF1a, HNF6, C/EBP etc...) are not affected while some typical differentiation markers are affected is puzzling. What could be the link between morphogenesis and the expression of the differentiation markers studied in the present paper ?

<u>Reviewer 2</u>

Advance summary and potential significance to field

Although the core developmental signaling pathways (e.g. Notch, TGFb, and Hippo) involved in regulating the bipotential hepatoblast progenitor cell fate choice between hepatocyte or cholangiocyte differentiation are beginning to be revealed the involvement of mechanotransduction in cell identity is unknown. In previous publications, Dr. Marino Zerial's group determined that hepatocytes assemble apical connections between juxtaposed hepatocytes (i.e., bulkheads) which

© 2024. Published by The Company of Biologists under the terms of the Creative Commons Attribution License (https://creativecommons.org/licenses/by/4.0/).

propagate anisotropic expansion generating tubular bile canaliculi instead of polarized lumenal duct-like structures. Importantly, Dr. Zerial's group has demonstrated that the small GTPase Rab35 is required in vitro and in vivo for correct self-organization of hepatoblasts into biaxial hepatocyte cell interactions forming bile canaliculi instead of vectorial duct-like epithelial tubes. The reduction of Rab35 in vivo mediated by lipid nanoparticle delivery of siRNA against Rab35 at E13.5 prior to establishing hepatoblast cell fate choice between hepatocyte or cholangiocyte differentiation results in duct-like structures throughout the parenchyma instead of proximal to the portal vein as expected for cholangiocyte specification and patent bile duct formation. The cells comprising the duct-like structure express the hepatocyte marker HNF4a but not the early specification cholangiocyte marker Sox9, suggesting that the cells of the duct-like structure in the absence of Rab35 have not entered the typical cholangiocyte transcriptional program.

This manuscript begins to address whether polarization and/or mechanotransduction including actomyosin membrane contractility or luminal pressure, fundamentally impacts bipotential hepatoblast progenitor differentiation and thereby the decision between the hepatocyte or cholangiocyte cell fate. The authors use an in vitro culture system wherein primary mouse hepatoblasts (E13.5) enter the hepatocyte differentiation program by generating multi-axial polarization and bile canaliculi. Importantly, the phenotype and morphology of hepatocyte bile canaliculi formation can be switched to vectoral duct-like phenotype upon knockdown of Rab35.

To add novelty beyond the authors previous publication, they demonstrate that the phenotypic switch is not specific to Rab35 but the molecular pathway required for the authors' described apical bulkhead formation including Dennd1b (Rab35 guanine-nucleotide exchange factor DENN domain containing 1B) and Acap2 (Rab35 effector ArfGAP with coiled-coil, ankyrin repeat and PH domains 2). With the advent of single cell RNA sequencing, temporal transcriptome assessment of hepatoblast differentiation suggests that the default cell decision of the hepatoblast is hepatocyte. Only hepatoblasts proximal to the portal vein begin to diverge from the default transcriptional program and upregulate cholangiocyte specific genes coincident with repression of the hepatocyte transcriptional program. Therefore the authors performed bulk RNA sequencing to determine the hepatocyte and cholangiocyte transcriptional programs comparing hepatoblasts undergoing bile canaliculi formation and the vectoral duct-like phenotype switch upon knockdown of Rab35. As expected, genes associated with the hepatocyte transcriptional program were decreased coincident with increase of genes in the cholangiocyte transcriptional program. Proteomic and immunofluorescence confirmed the RNA sequencing results.

Based on an assessment of select target genes for Notch, Tgfb and Hippo the phenotypic switch due to reduced Rab35 is not due to differential expression of these developmental pro-cholangiocyte pathways.

To further determine whether mechanotransduction is playing a role in the phenotypic switch in lumen growth (anisotropic vs. isotropic duct-like expansion), 1) contractility was reduced by knockdown of non-muscle myosin IIa (NMIIA, Myh9), and 2) luminal pressure was increased by polyethylene glycol 950 (PEG950) treatment to mediate water influx into bile canaliculi. In both cases the phenotypic switch in lumen growth was induced and expected changes in gene transcription was observed by qRT-PCR.

Comments for the author

Comments:

1. To understand the hepatocyte differentiation state, especially the interesting and critical phenotypic switch of HNF4a+ and Sox9- cells, it would strengthen to associate with functional landmark developmental transcript profiles (as an example, but any temporal published data set could be used, the Cytochrome P450 PMID: 22434873 as a comparison of hepatocyte cell maturation state).

2. Authors demonstrate cholangiocyte transcriptional program genes are increased coincident with down-regulation of hepatocyte associated genes at day 4 in culture and Rab35 siRNA hepatoblast transfection. This is one day after differences in lumen growth (anisotropic vs. isotropic duct-like expansion). Are the authors invoking a cholangiocyte signature, not yet identified, that normally exists prior to initiation of Sox9 or is an aberrant signature initiated in the

© 2024. Published by The Company of Biologists under the terms of the Creative Commons Attribution License (https://creativecommons.org/licenses/by/4.0/).

context of reduced Rab35 pathway? Can these cells fully enter the cholangiocyte transcriptional program or is this a dead-end cell state? Additionally, is this a cell state/transcriptional signature observed in published human data states or animal experimental models of human disease?

3. Given the different temporal transcriptional data sets, can a trajectory of cell state and transcriptional signature be identified upon knockdown of Rab35?

4. Moreover, as the authors discuss, bile ducts form through a primitive ductal structures (PDS) wherein transient asymmetric expression patterns are observed. Are any of the differentially expressed cholangiocyte genes identified in this study asymmetrically expressed in vivo in the transient PDS wherein HNF4a is still expressed but not all cholangiocyte identity genes are expressed.

5. This is a unique opportunity to identify genes that are expressed potentially due to mechanotransduction and not potentially dependent on chromatin remodeling or epigenetic modification to lineage switch between hepatocyte or cholangiocyte differentiation.

Reviewer 3

Advance summary and potential significance to field

The concept of morphogenesis related feed back control on gene regulatory pathways and cell fate decisions is important and less explored.In this paper, Lenka Belicova, et al have explored the relationship between cell related morphology and fate. They chose hepatocyte to cholangiocyte identity shift to test their hypothesis. They employed siRNA knockdown of Rab35 involved in controlling cytoskeletal regulation to showcase if by manipulating cell polarity, identity can shift from hepatocyte to cholangiocyte cell type.

Comments for the author

Comments:

1. In Figure 1D, no statistical analysis has been done. It is not clear if the difference observed between the siRNA knockdown and the control groups are statistically significant.

2. Line 100-109. Please add the function or relevance of genes so the reader has a better grasp of their importance in the context of hepatocyte vs biliary fates.

3. The hepatoblasts from E13.5 were isolated using the DLK1 antibody. Data on the purity of this isolation and culture images phase and IF should be provided. DLK1 also marks stellate cells in the liver. It is OK if the culture is not pure, but the data should be interpreted with this in mind and also clarified for readers. if the cultures contain stellate cells, would there be any non-cell autonomous effect from stellate cell function (co-culture)? As portal mesenchyme and other similar cell types within the liver (differentiated myofibroblasts) can send a key signal for cholangiocyte differentiation, this matter should be carefully assessed.

4. Please provide data that show siRNA-mediated knockdown has successfully downregulated the target gene. To make a more reliable conclusion about whether Rab35 and the other two proteins could be efficiently ablated, western blotting or other protein analysis techniques must be done to confirm the related protein level could be suppressed.

5. The authors conclude that the abrogation of hepatocyte identity pathways and shift to the cholanocyte identity result from switching the cellular polarization phenotype brought about by Rab35 knockdown. It is not quite clear how the authors rule out the possibility that Rab35 knockdown could result in the change in identity through another mechanism and, at the same time, due to its involvement in cytoskeletal regulation, has given rise to a change in cellular polarization phenotype as well. Does the fact that these two phenomena have been observed together imply the changing of the cellular polarization phenotype feeds back on cell identity pathways? Please clarify by data or discuss.

© 2024. Published by The Company of Biologists under the terms of the Creative Commons Attribution License (https://creativecommons.org/licenses/by/4.0/).

6. The data shown in Fig 3 (manipulation of actomyosin contractility and luminal pressure) , does not provide convincing evidence that changing the cell polarity can change cell identity as they have just tested a few marker genes. To further corroborate, it would be more convincing if they zero in on the GRNs involved and how they can change in response to manipulating cell polarity. They need to show a shift in GRNs governing hepatocyte identity to GRNs associated with cholangiocyte fate.

7. a larger area of cultural conditions should be provided for a better understanding of morphological features. Most images are substantially zoomed in.

8. SD or SEM on figures are mostly missing. In this regard, proper statistical analysis should be included.

First revision

Author response to reviewers' comments

Response to Reviewers

Reviewer 1 Advance Summary and Potential Significance to Field:

Earlier work from the authors (Belicova et al. J Cell Biol 2021) showed that upon silencing of the GTPase Rab35, multi-axial polarity of the hepatoblasts switches towards a vectorial apico-basal polarity. This is associated with loss of apical bulkheads and isotropic development of canaliculi which form cysts instead of tubes. It could not firmly be concluded from this work if loss of bulkheads perturbs the polarity or if perturbed polarity causes loss of bulkheads and cyst formation. The paper was subtly ambiguous about this issue and did not identify the primum movens, but nicely uncovered the existence of bulkheads. Differentiation of the hepatoblasts was not studied in depth.

Here the authors further investigate the consequences of Rab35 silencing in primary cultures of purified mouse hepatoblasts, and focus on the link between polarity and differentiation of the cells. Inhibition of Rab35 reduced expression of hepatocyte differentiation genes while stimulating the expression of markers of cholangiocyte differentiation and vectorial polarity. Changes in differentiation were preceded by isotropic expansion of the canaliculi, suggesting that differentiation is modulated by the apical morphology, not the opposite. This was further confirmed by relaxing subapical contractility (knockdown of non-muscle myosin IIa) or increasing intraluminal pressure with polyethylene glycol, both of which cause canalicular expansion and downregulation of hepatocyte genes. The authors conclude that cell polarity and lumen morphogenesis feedback to hepatocyte differentiation. Such feedback is conceptually novel in the field of liver development.

Reviewer 1 Comments for the Author:
The paper is clearly written and very nicely illustrated. The experiments are well designed and the conclusions are in general well supported by the data. The major weakness is in the lack of in vivo data, but the authors appropriately discuss the limitations of their experimental system.

The authors should address the following issues:

Figure 3 shows the expression of genes following knockdown of non-muscle myosin IIa and polyethylene glycol treatment. The number of tested genes is really low to support the conclusions. The authors should at least measure the 8 downregulated and 10 upregulated genes highlighted in Fig. 1B.

We thank the reviewer for raising this important point. We now performed bulk RNA-sequencing to analyse global changes in gene expression upon NMIIA knockdown and PEG950 treatment. We

© 2024. Published by The Company of Biologists under the terms of the Creative Commons Attribution License (https://creativecommons.org/licenses/by/4.0/).

find that both NMIIA knockdown and PEG950 treatment reduced the expression of multiple hepatocyte marker genes that were also downregulated upon Rab35 knockdown (Fig. 3E,I, File S5Table S6). Furthermore, KEGG pathway analysis of all downregulated genes revealed an enrichment of pathways associated with typical hepatocyte functions (Fig. 3F,J). These findings demonstrate that hepatoblast-to-hepatocyte differentiation is impaired following NMIIA knockdown and PEG950 treatment. In addition, NMIIA knockdown increased the expression of 32 out of the 81 genes that are upregulated upon Rab35 knockdown and enriched in cholangiocytes at E17.5 *in vivo* (Fig. 3E, Table S6). Interestingly, NMIIA knockdown also significantly increased the expression of bona fide cholangiocyte markers, e.g. Sox4, Sox9, Hes1. The effect of PEG950 on cholangiocyte-associated genes was less strong, upregulating 8 out of the 81 genes that are upregulated upon Rab35 knockdown and enriched in E17.5 cholangiocytes (Table S6). This may be due to the shorter duration of this treatment as compared to Rab35 and NMIIA knockdown. We stimulated the cells with PEG950 at day 3, after the initial formation of nascent BC, to limit toxic effects of prolonged PEG950 treatment and avoid interfering with initial apical lumen formation. In the revised manuscript, we reported the new RNA sequencing results and updated the conclusions (lines 199-225).

Point of discussion. Could the delay between morphological changes (cyst formation) and transcriptional changes following knockdown of Rab35 result from the fact that polarity changes occur faster than transcriptional changes, with polarity being - for instance - dependent on fast and direct changes in protein activity induced by Rab35 knockdown, and transcriptional changes being secondary to slower processes requiring Rab35-dependent changes in transcription factor activity and slow control of transcription? In that case, there would be no need to consider that morphogenesis is the inducer of transcriptional changes; both morphogenesis and differentiation would be dependent on Rab35, the one being faster than the other.

We agree with the reviewer that the delay between morphological changes (cyst formation) and transcriptional changes following Rab35 knockdown alone cannot prove a causal relationship between these two processes. However, the sporadic expression of Trop2 in control cells with enlarged apical lumina suggest that changes in apical lumen morphology (i.e. enlarged, more spherical, lumina instead of tubular BC) can affect the gene expression profile of differentiating hepatoblasts even in the presence of Rab35. Therefore, to test whether apical lumen morphology influences hepatocyte differentiation, we perturbed apical lumen morphology using two independent methods, 1) by reducing the contractility of the subapical actomyosin cortex and 2) by increasing luminal pressure (Fig. 3). Similar to Rab35 knockdown, both perturbations impaired hepatocyte differentiation, and NMIIA knockdown also increased the expression of several cholangiocyte-associated genes. Combined, these results demonstrate that the induction of isotropic apical lumen expansion, instead of tubular BC elongation, impairs hepatoblast-to-hepatocyte differentiation, and suggest a link between isotropic lumen expansion and hepatoblast-to-cholangiocyte differentiation, see lines 184-190 and lines 218-225 in the revised document.

Point of discussion. The fact that transcription factors of the core cholangiocyte and hepatocyte gene regulatory networks (SOX9, SOX4, HNF1b, HNF4, HNF1a, HNF6, C/EBP, etc...) are not affected while some typical differentiation markers are affected is puzzling. What could be the link between morphogenesis and the expression of the differentiation markers studied in the present paper?

Our data showing lack of activation of Notch, TGFb, and Hippo target genes argue that the transcriptional regulation upon changes in cell polarity and apical lumen morphology is more complex. To identify the transcription factors that could link changes in apical lumen morphology to changes in gene expression, we analysed the RNA sequencing data for transcription factors that were associated with hepatocytes or cholangiocytes isolated from mouse livers at E17.5 (Yang et al. 2023) and were differentially regulated in response to Rab35 knockdown, NMIIA knockdown, and PEG950 treatment. We identified three hepatocyte- enriched transcription factors (Bhlha15/Mist1, Klf12, Nr1i3) that were downregulated in all conditions.
Interestingly, Bhlha15/Mist1 and Nr1i3 have previously been linked to hepatoblast-to-hepatocyte differentiation and Cyp3a11 expression (Chikada et al., 2015, Wei et al., 2002). Furthermore, we observed upregulation of the cholangiocyte-enriched transcription factors Lin28a, Grhl1 and

© 2024. Published by The Company of Biologists under the terms of the Creative Commons Attribution License (https://creativecommons.org/licenses/by/4.0/).

Grhl2, as well as several Grhl2 target genes, upon Rab35 and NMIIA knockdown. These transcription factors are thus candidate players that may link between morphogenesis and the transcriptional changes observed for the differentiation markers. We have included these findings in the revised manuscript (lines 230-238).

Reviewer 2 Advance Summary and Potential Significance to Field:

Although the core developmental signaling pathways (e.g. Notch, TGFb, and Hippo) involved in regulating the bipotential hepatoblast progenitor cell fate choice between hepatocyte or cholangiocyte differentiation are beginning to be revealed, the involvement of mechanotransduction in cell identity is unknown. In previous publications, Dr. Marino Zerial's group determined that hepatocytes assemble apical connections between juxtaposed hepatocytes (i.e., bulkheads) which propagate anisotropic expansion generating tubular bile canaliculi instead of polarized lumenal duct-like structures. Importantly, Dr. Zerial's group has demonstrated that the small GTPase Rab35 is required in vitro and in vivo for correct self-organization of hepatoblasts into biaxial hepatocyte cell interactions forming bile canaliculi instead of vectorial duct-like epithelial tubes. The reduction of Rab35 in vivo mediated by lipid nanoparticle delivery of siRNA against Rab35 at E13.5 prior to establishing hepatoblast cell fate choice between hepatocyte or cholangiocyte differentiation results in duct-like structures throughout the parenchyma instead of proximal to the portal vein as expected for cholangiocyte specification and patent bile duct formation. The cells comprising the duct-like structure express the hepatocyte marker HNF4a but not the early specification cholangiocyte marker Sox9, suggesting that the cells of the duct-like structure in the absence of Rab35 have not entered the typical cholangiocyte transcriptional program.

This manuscript begins to address whether polarization and/or mechanotransduction, including actomyosin membrane contractility or luminal pressure, fundamentally impacts bipotential hepatoblast progenitor differentiation and thereby the decision between the hepatocyte or cholangiocyte cell fate. The authors use an in vitro culture system wherein primary mouse hepatoblasts (E13.5) enter the hepatocyte differentiation program by generating multi-axial polarization and bile canaliculi. Importantly, the phenotype and morphology of hepatocyte bile canaliculi formation can be switched to vectoral duct-like phenotype upon knockdown of Rab35.

To add novelty beyond the authors previous publication, they demonstrate that the phenotypic switch is not specific to Rab35 but the molecular pathway required for the authors' described apical bulkhead formation including Dennd1b (Rab35 guanine-nucleotide exchange factor DENN domain containing 1B) and Acap2 (Rab35 effector ArfGAP with coiled-coil, ankyrin repeat and PH domains 2). With the advent of single cell RNA sequencing, temporal transcriptome assessment of hepatoblast differentiation suggests that the default cell decision of the hepatoblast is hepatocyte. Only hepatoblasts proximal to the portal vein begin to diverge from the default transcriptional program and upregulate cholangiocyte specific genes coincident with repression of the hepatocyte transcriptional program. Therefore, the authors performed bulk RNA sequencing to determine the hepatocyte and cholangiocyte transcriptional programs comparing hepatoblasts undergoing bile canaliculi formation and the vectoral duct-like phenotype switch upon knockdown of Rab35. As expected, genes associated with the hepatocyte transcriptional program were decreased coincident with increase of genes in the cholangiocyte transcriptional program. Proteomic and immunofluorescence confirmed the RNA sequencing results.

Based on an assessment of select target genes for Notch, Tgfb and Hippo the phenotypic switch due to reduced Rab35 is not due to differential expression of these developmental pro-cholangiocyte pathways. To further determine whether mechanotransduction is playing a role in the phenotypic switch in lumen growth (anisotropic vs. isotropic duct-like expansion), 1) contractility was reduced by knockdown of non-muscle myosin IIa (NMIIA, Myh9), and 2) luminal pressure was increased by polyethylene glycol 950 (PEG950) treatment to mediate water influx into bile canaliculi. In both cases the phenotypic switch in lumen growth was induced and expected changes in gene transcription was observed by qRT-PCR.

Reviewer 2 Comments for the
Author: Comments:

© 2024. Published by The Company of Biologists under the terms of the Creative Commons Attribution License (https://creativecommons.org/licenses/by/4.0/).

1. To understand the hepatocyte differentiation state, especially the interesting and critical phenotypic switch of HNF4a+ and Sox9- cells, it would strengthen to associate with functional landmark developmental transcript profiles (as an example, but any temporal published data set could be used, the Cytochrome P450 PMID: 22434873 as a comparison of hepatocyte cell maturation state).

We thank the reviewer for this helpful suggestion. We cross-referenced our time-course RNA sequencing data with published temporal bulk RNA sequencing data from mouse developing liver (Yang et al., 2023, available under GSE142089). This dataset contains mRNA expression profiles of FACS-sorted liver cells based on the surface markers Dlk1 (hepatoblasts and fetal hepatocytes) and EpCAM (fetal cholangiocytes), from E12.5 to E17.5. This time frame overlaps with day 0 (day of hepatoblast isolation at E13.5) – day 4 in our *in vitro* culture. In siCtrl-transfected cells *in vitro*, the expression levels of the hepatocyte landmark genes *Alb*, *Cyp3a11*, *Serpina1b* and *Trf* increase over time (Fig. 2C), which is in line with the transcriptional increase of these genes in hepatocytes, but not cholangiocytes, from E15.5 *in vivo* (Fig. S2A).

In the first version of the manuscript, we described our *in vitro* hepatoblast culture system modelling prenatal hepatoblast-to-hepatocyte differentiation (Belicova et al., 2021, JCB) and used the word 'mature' to describe the *in vitro*-differentiated hepatocytes expressing typical hepatocyte genes, such as *Cyp3a11* and *Alb*. However, in light of the comment from the reviewer, we think that it is more correct to state that these cells are foetal hepatocytes and not yet mature hepatocytes. We have therefore adapted this in the revised manuscript (line 152).

2. Authors demonstrate cholangiocyte transcriptional program genes are increased coincident with down- regulation of hepatocyte associated genes at day 4 in culture and Rab35 siRNA hepatoblast transfection. This is one day after differences in lumen growth (anisotropic vs. isotropic duct-like expansion). Are the authors invoking a cholangiocyte signature, not yet identified, that normally exists prior to initiation of Sox9 or is an aberrant signature initiated in the context of reduced Rab35 pathway? Can these cells fully enter the cholangiocyte transcriptional program or is this a dead-end cell state?

Our results demonstrate that isotropic expansion of the apical lumen upon Rab35 knockdown impairs hepatoblast-to-hepatocyte differentiation and upregulates a number of cholangiocyte-associated genes, raising the question as to whether mechanosensing of apical lumen morphology contributes to cholangiocyte differentiation in asymmetric bile ducts *in vivo* (discussed in lines 218-225, 257-261). Previous studies have demonstrated that signalling pathways (e.g. Hippo, Notch and Tgf-□) also contribute to cholangiocyte differentiation during this developmental stage (line 243-247). However, knockdown of Rab35 changes apical lumen morphology, but does not activate such signalling pathways (Fig. 2E). For this reason, we do not expect that the cholangiocyte signature of siRab35-transfected cells is a separate developmental stage prior to Sox9 expression. Our interpretation is that the cells in our *in vitro* culture cannot fully enter the cholangiocyte transcriptional program. They may over time, except that our primary hepatoblasts culture are only viable for up to 6 days. This interpretation is in line with published single-cell RNA sequencing data of the developing liver (Yang et al., 2017, GSE90047), where we could not detect Grhl2-positive Sox9-negative cells. Furthermore, temporal bulk RNA sequencing (Yang et al., 2023, GSE142089) revealed similar temporal expression patterns of Grhl2 and Sox9 in Epcam-positive foetal cholangiocytes.

Additionally, is this a cell state/transcriptional signature observed in published human data states or animal experimental models of human disease?

Recently, we observed the presence of abnormally dilated bile canaliculi in liver tissue of patients affected by primary sclerosing cholangitis, resembling the liver cell rosettes described in other liver diseases (Mayer et al., 2023). Similar to siRab35-transfected hepatoblasts, the hepatocytes in these rosettes face an enlarged apical lumen and display vectorial polarity. Interestingly, some of these cells express low levels of cholangiocyte markers, e.g. Sox9, suggesting there may be a feedback of cell polarity and lumen morphology on cellular (de)differentiation in these rosettes. However, since the cells in these structures only minimally express typical cholangiocyte or

© 2024. Published by The Company of Biologists under the terms of the Creative Commons Attribution License (https://creativecommons.org/licenses/by/4.0/).

hepatobiliary markers, it is difficult to identify them in publicly available single-cell RNA-seq data, which typically suffer from low recovery of cholangiocyte and hepatobiliary cells, or low gene recovery. The link between apical lumen morphology and cell fate in the context of liver disease is the subject of ongoing investigations in our lab.

3. Given the different temporal transcriptional data sets, can a trajectory of cell state and transcriptional signature be identified upon knockdown of Rab35?

To identify genes that drive the transcriptional signature upon Rab35 knockdown, we used correspondence analysis (Gralinska and Vingron, 2023) of the time-course RNA sequencing data (File S1S3). This analysis shows similar trajectories of siCtrl- and siRab35-transfected cells at Day 1-3, and a bifurcation at Day 4 when gene expression changes in siRab35-transfected cells. We then used an association plot to determine genes that are specific to Day 4 and Day 5 in siRab35 samples (Fig. 2D, Table S5) and find the same cholangiocyte-associated genes as identified in Fig. 1B, e.g. *Grhl2*, *Ap1m2*, *Rab25*, *Cav1*.

4. Moreover, as the authors discuss, bile ducts form through a primitive ductal structures (PDS) wherein transient asymmetric expression patterns are observed. Are any of the differentially expressed cholangiocyte genes identified in this study asymmetrically expressed in vivo in the transient PDS wherein HNF4a is still expressed but not all cholangiocyte identity genes are expressed.

We expect that the mechano-sensing and -transduction pathways responding to changes in apical lumen morphology act simultaneously with signalling pathways (e.g. Notch, Hippo, Tgf-□) to drive cholangiocyte differentiation. Hence, we did not expect to find HNF4□-positive/Sox9-negative cells that express the cholangiocyte-associated genes identified upon Rab35 knockdown. However, to test whether we could find such cells in asymmetric bile ducts, we stained embryonic liver tissue at E15.5 for Sox9 in combination with Trop2 and Cav1. Despite several attempts with multiple staining protocols, we did not manage to convincingly stain Trop2 and Cav1, even in Sox9-positive cells.

5. This is a unique opportunity to identify genes that are expressed potentially due to mechanotransduction and not potentially dependent on chromatin remodeling or epigenetic modification to lineage switch between hepatocyte or cholangiocyte differentiation.

We identified 32 genes, including the transcription factor Grhl2 and several of its target genes, that are upregulated following Rab35 knockdown and NMIIA knockdown and are enriched in cholangiocytes at E17.5 *in vitro* (Table S6). We therefore suspect that these genes are potentially upregulated in response to mechanotransduction pathways during cholangiocyte differentiation. However, we cannot exclude that chromatin remodelling or epigenetic modifications are involved in the regulation of these genes, especially since multiple studies demonstrate that mechanical stimulation can result in chromatin remodelling and epigenetic modifications (Dupont et al., 2022; Song et al., 2022).

Reviewer 3 Advance Summary and Potential Significance to Field:

The concept of morphogenesis related feed back control on gene regulatory pathways and cell fate decisions is important and less explored. In this paper, Lenka Belicova, et al have explored the relationship between cell related morphology and fate. They chose hepatocyte to cholangiocyte identity shift to test their hypothesis. They employed siRNA knockdown of Rab35 involved in controlling cytoskeletal regulation to showcase if by manipulating cell polarity, identity can shift from hepatocyte to cholangiocyte cell type.

Reviewer 3 Comments for the Author:

Comments:

1. In Figure 1D, no statistical analysis has been done. It is not clear if the difference observed between the siRNA knockdown and the control groups are statistically significant.

© 2024. Published by The Company of Biologists under the terms of the Creative Commons Attribution License (https://creativecommons.org/licenses/by/4.0/).

We have now added the statistical analysis to Fig. 1D. For all proteins, the differences between the Rab35 knockdown and control groups were statistically significant, with the exception of Cyp3a11, because its expression was below the detection limit in three out of four samples of the siRab35 condition, whereas it was detected in all four samples in the control conditions.

2. Line 100-109. Please add the function or relevance of genes so the reader has a better grasp of their importance in the context of hepatocyte vs biliary fates.

We completely agree with the reviewer that adding the function or relevance of the genes would help the reader. In view of the limited word count in the main text that is accepted for a short report, we have added the function and relevance of these genes in a separate table (Table S1).

3. The hepatoblasts from E13.5 were isolated using the DLK1 antibody. Data on the purity of this isolation and culture images phase and IF should be provided. DLK1 also marks stellate cells in the liver. It is OK if the culture is not pure, but the data should be interpreted with this in mind and also clarified for readers. if the cultures contain stellate cells, would there be any non-cell autonomous effect from stellate cell function (co-culture)? As portal mesenchyme and other similar cell types within the liver (differentiated myofibroblasts) can send a key signal for cholangiocyte differentiation, this matter should be carefully assessed.

Immunofluorescent staining indeed revealed the presence of small numbers of Desmin-positive cells (stellate cells and other mesenchymal cells) in the *in vitro* culture. Given the role of Jagged-1-expressing portal mesenchyme in hepatoblast biliary fate specification via Notch signalling (Zong et al., 2009, Hofmann et al., 2010), we investigated whether cell-cell contacts between mesenchymal cells and hepatoblasts are responsible for the upregulation of cholangiocyte-associated genes upon Rab35 silencing. However, we found that the majority of Trop2-positive hepatoblasts in the cysts are not in contact with mesenchymal cells, arguing that at least such cell-cell contacts do not account for the observed phenotype. We now added these data to the revised manuscript (lines 127-132, Fig. S1E).

4. Please provide data that show siRNA-mediated knockdown has successfully downregulated the target gene. To make a more reliable conclusion about whether Rab35 and the other two proteins could be efficiently ablated, western blotting or other protein analysis techniques must be done to confirm the related protein level could be suppressed.

The Rab35 knockdown efficiency on the protein level has already been carefully demonstrated in Fig. 5D of our previous paper (Belicova et al., 2021, JCB). Furthermore, the time-course RNA sequencing data shows the silencing of Rab35 on the mRNA level on each day of the culture (Fig. 2B). To make this point clear we now added the reference to this paper in line 88.

Currently, we could not find good antibodies for Acap2 and Dennd1b. Therefore, we confirmed the silencing of these genes on the mRNA level (Fig. S1B).

5. The authors conclude that the abrogation of hepatocyte identity pathways and shift to the cholanocyte identity result from switching the cellular polarization phenotype brought about by Rab35 knockdown. It is not quite clear how the authors rule out the possibility that Rab35 knockdown could result in the change in identity through another mechanism and, at the same time, due to its involvement in cytoskeletal regulation, has given rise to a change in cellular polarization phenotype as well. Does the fact that these two phenomena have been observed together imply the changing of the cellular polarization phenotype feeds back on cell identity pathways? Please clarify by data or discuss.

Please see our response to comment 2 of Reviewer 1. We now discuss this in the revised document lines 184- 190 and lines 218-225.

6. The data shown in Fig 3 (manipulation of actomyosin contractility and luminal pressure) , does not provide convincing evidence that changing the cell polarity can change cell identity as they have just tested a few marker genes. To further corroborate, it would be more convincing if they zero in on the GRNs involved and how they can change in response to manipulating cell polarity.

© 2024. Published by The Company of Biologists under the terms of the Creative Commons Attribution License (https://creativecommons.org/licenses/by/4.0/).

They need to show a shift in GRNs governing hepatocyte identity to GRNs associated with cholangiocyte fate.

We agree with the reviewer and performed bulk RNA-sequencing to analyse global changes in gene expression upon NMIIA knockdown and PEG950 treatment (see reply to the first comment of Reviewer 1). Similar to Rab35 knockdown, NMIIA knockdown and PEG950 treatment did not affect the expression of major hepatocyte- associated transcription factors, e.g. Hnf4a, Cebpa and Prox1, even though all three conditions cause downregulation of hepatocyte-associated genes. In our data, we found three hepatocyte-associated transcription factors (Bhlha15/Mist1, Klf12, Nr1i3) that were downregulated in response to Rab35 knockdown, NMIIA knockdown and PEG950 treatment. Interestingly, Bhlha15/Mist1 has previously been linked to hepatoblast-to- hepatocyte differentiation and Cyp3a11 expression (Chikada et al., 2015, Wei et al., 2002). We also found upregulation of the cholangiocyte-enriched transcription factors *Lin28a*, *Grhl1* and *Grhl2*, as well as several Grhl2 target genes, in response to Rab35 and NMIIA knockdown. We have included this analysis in the revised manuscript (lines 230-238).

7. a larger area of cultural conditions should be provided for a better understanding of morphological features. Most images are substantially zoomed in.

We thank the reviewer for this suggestion. We have now added larger field-of-view images for siCtrl, siRab35, siDennd1b and siAcap2 in Fig. S1A, and for siNMIIA and PEG950-treatment in Fig. S4.

8. SD or SEM on figures are mostly missing. In this regard, proper statistical analysis should be included.

We agree with the reviewer and included statistical analyses in Fig. 1D, Fig. 2B,C,E, Fig. S2A,B, and Fig. S4A of the revised manuscript. We have also added the SD in Fig. 1D and Fig. S4A. We believe that adding the SD to the graphs of the time-course RNAseq experiment, which have only three data points, makes the graph less clear (see Rebuttal Fig. 1). However, if the reviewer and/or editor prefer, we will add the SD to the time-course RNAseq graphs.

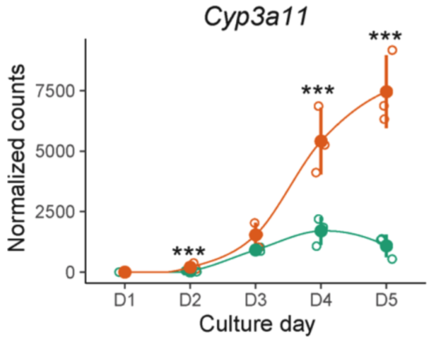

Rebuttal Fig. 1 Expression profile *Cyp3a11* in siCtrl- and siRab35-transfected cells. Data points represent normalized counts of three biological repeats. Error bars represent SD. ***, p-adjusted < 0.001.

Second decision letter

MS ID#: DEVELOP/2024/202777

MS TITLE: Hepatocyte differentiation requires anisotropic expansion of bile canaliculi

AUTHORS: Maarten Paul Bebelman, Lenka Belicova, Elzbieta Gralinska, Tobias Jumel, Aparajita Lahree, Sarah Sommer, Andrej Paul Shevchenko, Timofei Zatsepin, Yannis Kalaidzidis, Martin Vingron, and Marino Zerial
ARTICLE TYPE: Research Report

© 2024. Published by The Company of Biologists under the terms of the Creative Commons Attribution License (https://creativecommons.org/licenses/by/4.0/).

I am happy to tell you that your manuscript has been accepted for publication in Development, pending our standard publication integrity checks.

Reviewer 1

Advance summary and potential significance to field

Earlier work from the authors (Belicova et al. J Cell Biol 2021) showed that upon silencing of the GTPase Rab35, multi-axial polarity of the hepatoblasts switches towards a vectorial apico-basal polarity. This is associated with loss of apical bulkheads and isotropic development of canaliculi which form cysts instead of tubes. It could not firmly be concluded from this work if loss of bulkheads perturbs the polarity or if perturbed polarity causes loss of bulkheads and cyst formation. The paper was subtly ambiguous about this issue and did not identify the primum movens, but nicely uncovered the existence of bulkheads. Differentiation of the hepatoblasts was not studied in depth.

Here the authors further investigate the consequences of Rab35 silencing in primary cultures of purified mouse hepatoblasts, and focus on the link between polarity and differentiation of the cells. Inhibition of Rab35 reduced expression of hepatocyte differentiation genes while stimulating the expression of markers of cholangiocyte differentiation and vectorial polarity. Changes in differentiation were preceded by isotropic expansion of the canaliculi, suggesting that differentiation is modulated by the apical morphology, not the opposite. This was further confirmed by relaxing subapical contractility (knockdown of non-muscle myosin IIa) or increasing intraluminal pressure with polyethylene glycol both of which cause canalicular expansion and downregulation of hepatocyte genes. The authors conclude that cell polarity and lumen morphogenesis feedback to hepatocyte differentiation. Such feedback is conceptually novel in the field of liver development.

Comments for the author

The reviewer thanks the authors for having considered all comments with care. The manuscript is very significantly improved.

Reviewer 2

Advance summary and potential significance to field

Although the core developmental signaling pathways (e.g. Notch, TGFb, and Hippo) involved in regulating the bipotential hepatoblast progenitor cell fate choice between hepatocyte or cholangiocyte differentiation are beginning to be revealed, the involvement of mechanotransduction in cell identity is unknown.

This manuscript begins to address whether polarization and/or mechanotransduction, including actomyosin membrane contractility or luminal pressure, fundamentally impacts bipotential hepatoblast progenitor differentiation and thereby the decision between the hepatocyte or cholangiocyte cell fate. The authors use an in vitro culture system wherein primary mouse hepatoblasts (E13.5) enter the hepatocyte differentiation program by generating multi-axial polarization and bile canaliculi. Importantly, the phenotype and morphology of hepatocyte bile canaliculi formation can be switched to vectoral duct-like phenotype upon knock-down of Dennd1b (Rab35 guanine-nucleotide exchange factor DENN domain containing 1B) and Acap2 (Rab35 effector ArfGAP with coiled-coil, ankyrin repeat and PH domains 2). Based on an assessment of select target genes for Notch, Tgfb and Hippo the phenotypic switch due to reduced Rab35 is not due to differential expression of these developmental pro-cholangiocyte pathways.

Comments for the author

All comments and concerns have been addressed in the revision.

© 2024. Published by The Company of Biologists under the terms of the Creative Commons Attribution License (https://creativecommons.org/licenses/by/4.0/).

Reviewer 3

Advance summary and potential significance to field

as described before in initial review.

Comments for the author

the current version has addressed my concerns successfully.

© 2024. Published by The Company of Biologists under the terms of the Creative Commons Attribution License (https://creativecommons.org/licenses/by/4.0/).
